# A quick aphasia battery for efficient, reliable, and multidimensional assessment of language function

**DOI:** 10.1371/journal.pone.0192773

**Published:** 2018-02-09

**Authors:** Stephen M. Wilson, Dana K. Eriksson, Sarah M. Schneck, Jillian M. Lucanie

**Affiliations:** 1 Department of Hearing and Speech Sciences, Vanderbilt University Medical Center, Nashville, Tennessee, United States of America; 2 Department of Speech, Language, and Hearing Sciences, University of Arizona, Tucson, Arizona, United States of America; University of Zurich, SWITZERLAND

## Abstract

This paper describes a quick aphasia battery (QAB) that aims to provide a reliable and multidimensional assessment of language function in about a quarter of an hour, bridging the gap between comprehensive batteries that are time-consuming to administer, and rapid screening instruments that provide limited detail regarding individual profiles of deficits. The QAB is made up of eight subtests, each comprising sets of items that probe different language domains, vary in difficulty, and are scored with a graded system to maximize the informativeness of each item. From the eight subtests, eight summary measures are derived, which constitute a multidimensional profile of language function, quantifying strengths and weaknesses across core language domains. The QAB was administered to 28 individuals with acute stroke and aphasia, 25 individuals with acute stroke but no aphasia, 16 individuals with chronic post-stroke aphasia, and 14 healthy controls. The patients with chronic post-stroke aphasia were tested 3 times each and scored independently by 2 raters to establish test-retest and inter-rater reliability. The Western Aphasia Battery (WAB) was also administered to these patients to assess concurrent validity. We found that all QAB summary measures were sensitive to aphasic deficits in the two groups with aphasia. All measures showed good or excellent test-retest reliability (overall summary measure: intraclass correlation coefficient (ICC) = 0.98), and excellent inter-rater reliability (overall summary measure: ICC = 0.99). Sensitivity and specificity for diagnosis of aphasia (relative to clinical impression) were 0.91 and 0.95 respectively. All QAB measures were highly correlated with corresponding WAB measures where available. Individual patients showed distinct profiles of spared and impaired function across different language domains. In sum, the QAB efficiently and reliably characterized individual profiles of language deficits.

## Introduction

This paper describes a quick aphasia battery (QAB) that was designed to support research into neuroplasticity of language networks after damage to language regions of the brain. There are three crucial features needed in an aphasia battery in this research context.

First, it must be time-efficient and easily administered at the bedside. Many of the patients who are relevant to this kind of research, such as acute stroke patients and post-surgical patients, will not tolerate lengthy assessments [1]. Based on our practical experiences working with acute stroke patients, we sought to design a battery that could be administered to most patients in a quarter of an hour or less.

Second, the battery must be psychometrically sound [2]. It must have good inter-rater reliability, and good test-retest reproducibility so that changes due to recovery can be distinguished from changes due to measurement error. It should exhibit concurrent validity with respect to more comprehensive aphasia batteries that take longer to administer.

Third, it should yield a multidimensional characterization of impaired and spared aspects of language function. The concept of distinct aphasia subtypes (e.g. Broca’s aphasia, Wernicke’s aphasia) is valuable and has facilitated the development of an explanatory and enduring model of language organization in the brain [3–5]. However, many patients cannot be classified neatly as one subtype or another [6]. In reality, most individual cases of aphasia are the consequence of varying degrees of damage to multiple independent but interacting language subsystems with distinct but overlapping neural substrates. Accordingly, each case of aphasia should be characterized not as one of a number of discrete types, but rather as a point in a multidimensional symptom space [7–11]. The specific dimensions defining this space should, as far as possible, reflect functions that have been empirically demonstrated to be neurally and functionally distinct. Therefore, for instance, single word comprehension and sentence comprehension need to be quantified separately, since these can readily dissociate [12,13], and the multifaceted concept of non-fluency should be parcellated into different aspects such as word finding, grammatical construction, and motor speech [8,11,14–16].

To our knowledge, there are no existing English language aphasia batteries that meet these three criteria. Currently, the most widely used English language comprehensive batteries are the Boston Diagnostic Aphasia Exam (BDAE) [17], the Western Aphasia Battery (WAB) [18], and the Comprehensive Aphasia Test (CAT) [19]; see [20] for review of these and other tests. These batteries have been thoroughly validated, and provide a comprehensive assessment of an individual’s strengths and weaknesses in multiple language domains. However, they take from 30 minutes to several hours to administer. The BDAE and WAB have short forms, but the short form of the BDAE still takes 40 to 60 minutes to administer, while the short form of the WAB has not been validated [21].

As for aphasia tests that are quicker to administer, there are several rapid screening instruments that take only a few minutes, including the Frenchay Aphasia Screening Test [22], the speech/language sections of the NIH Stroke Scale [23], the Language Screening Test [24], and the Aphasia Rapid Test [25]; see [21] for review. These tests are quite effective for detecting the presence of aphasia and quantifying its severity. However due to their brevity they generally provide limited information regarding patients’ strengths and weaknesses in particular language domains. The Kentucky Aphasia Test [1] is a slightly longer but still brief battery that is designed to be clinician-friendly, and yields a set of subscores spanning core language domains. But only preliminary validation has been carried out, and the authors have described a need to adjust some of the items. The Bedside Evaluation Screening Test [26] is similar, consisting of several subtests in different domains and taking about 20 minutes to administer. However it includes minimal assessment of expressive syntax, receptive syntax, or connected speech, and a factor analysis showed that all items loaded on a single factor, suggesting little ability to differentiate between different aphasia profiles. Short forms of the Porch Index of Communicative Ability (PICA) [27] have been described and validated [28–31]. In particular, two different short forms have been described that can each be administered in about a quarter of an hour [29]. However the PICA, in standard or short form, is selective in the language functions sampled (e.g. there is no assessment of connected speech, and minimal assessment of sentence comprehension), and its factor structure is driven by a general language factor and input/output modalities more so than actual linguistic domains [32–34].

There is, therefore, a need for a new aphasia battery to fill the gap between comprehensive batteries and screening tests, to optimize assessment of aphasia in research contexts in which time is limited. In developing the QAB, design decisions were driven by the three goals described above. To assess multiple domains of language function, multiple subtests are required. But to keep the battery quick to administer, each subtest can contain only a limited number of items, so it is important to maximize the informativeness of each item. To that end, items were carefully chosen to span a wide difficulty range, so that mild as well as severe deficits can be quantified effectively, and a graded scoring system was developed so that information can be gleaned from responses that are neither completely correct nor completely incorrect. In order to reduce administration time, reading is assessed only in one respect (reading aloud), while writing is not assessed at all. In a clinical context it would be critical to assess these modalities, but in a research context, this was a necessary trade-off.

Several practical considerations were also taken into account. To make the QAB easier to administer at the bedside, especially in acute care settings, it requires only a score-sheet and a stimulus book. To make the QAB more appropriate for longitudinal studies, three equivalent forms were constructed, such that most of the items differ between forms, while being matched on relevant psycholinguistic properties. This reduces any item-specific learning that might take place. To remove barriers to adoption of cost or convenience, all materials have been made freely available along with this open access publication (S1 Test materials, S2 Test materials, S1 Macro).

## Material and methods

### Rationale, construction, administration and scoring of the QAB

**Fig 1:**
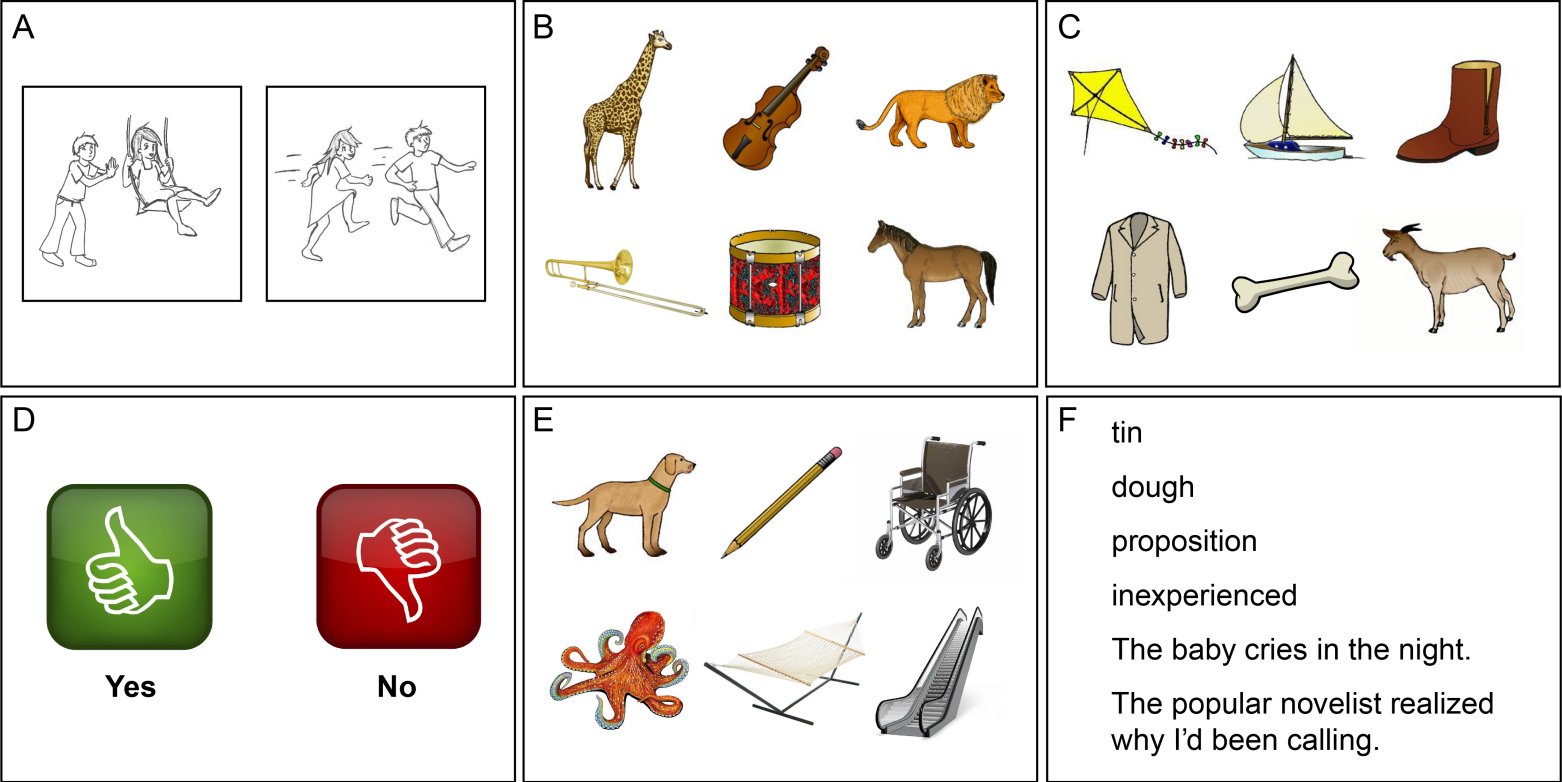
Sample stimulus cards from the QAB. (A) Action pictures for elicitation of connected speech. (B) Single word comprehension with semantic distractors. (C) Single word comprehension with phonemic distractors. (D) ‘Yes’ and ‘no’. (E) Picture naming. (F) Reading aloud.

The QAB includes eight subtests: (1) Level of consciousness; (2) Connected speech; (3) Word comprehension; (4) Sentence comprehension; (5) Picture naming; (6) Repetition; (7) Reading aloud; and (8) Motor speech. Each subtest contains between 5 and 12 items, each of which is scored on a 5-point scale running from 0 to 4. The precise meanings of the points on the scale vary depending on the item, and are indicated on the score-sheet. The QAB scoring system is simple enough that it can be scored online, but for research contexts it is recommended that the evaluation be recorded (audio, or better still, audiovisual) so that scores can be checked offline to maximize accuracy. The score-sheet for one form of the QAB is presented piece by piece in this paper; the score-sheets for all three forms are provided as supporting information (S1 Test materials). The stimulus cards for the first form are shown in Fig 1, and the stimulus cards for each of the three forms are provided as supporting information (S2 Test materials).

After completing the evaluation, eight summary measures are derived, as described below. The summary measures provide an individual profile of spared and impaired language domains.

### Subtest 1: Level of consciousness

**Fig 2:**
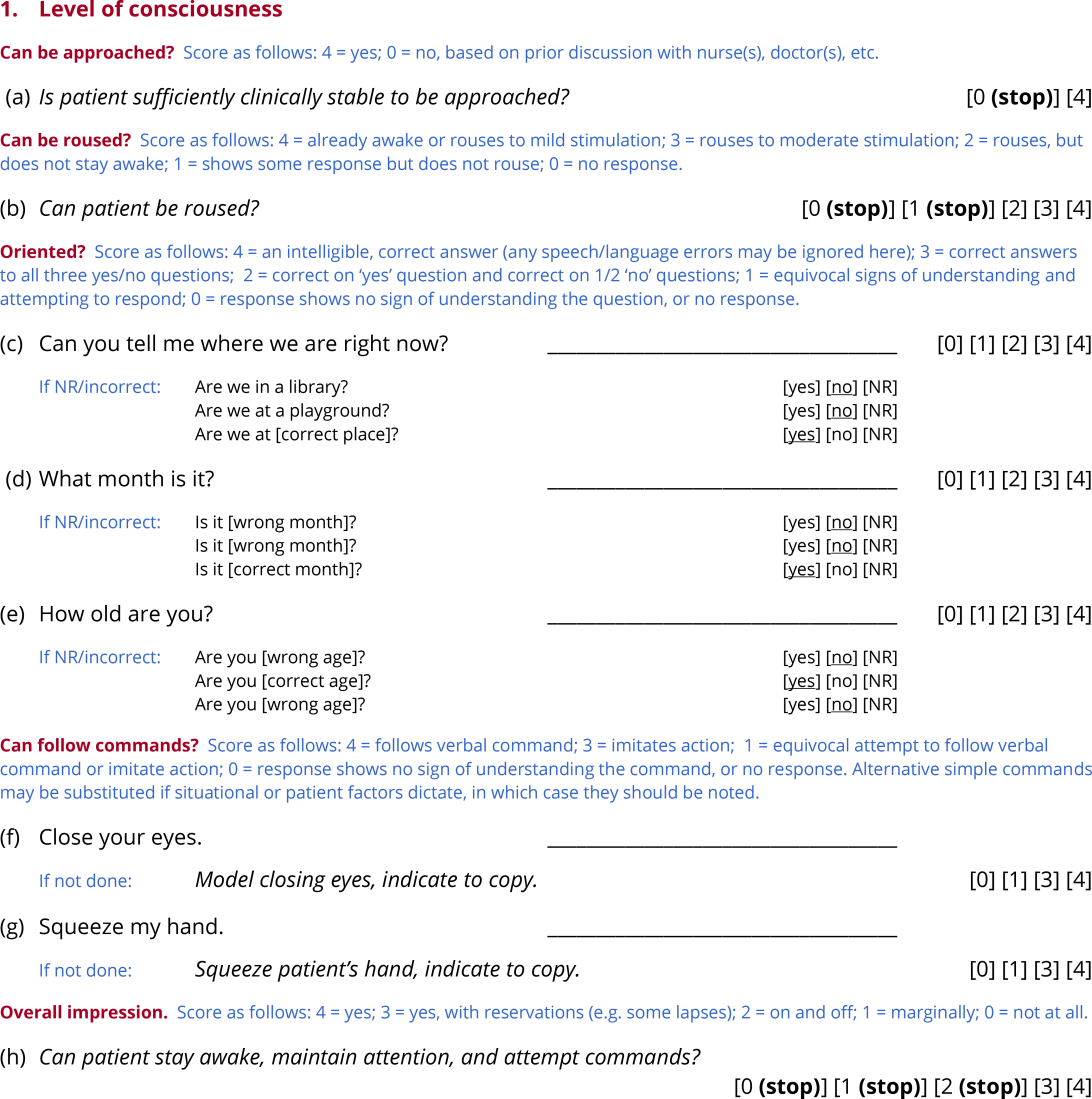
Subtest 1: Level of consciousness.

The purpose of the first subtest of the QAB is to determine whether it is in fact feasible to evaluate language function (Fig 2). This subtest is based on the level of consciousness section of the NIH stroke scale [23]. It is intended for situations in which a researcher is entering a hospital room, never before having met the patient they intend to evaluate, and potentially knowing little or nothing about the status of the patient. If the QAB is being used in a different context, such as a follow-up visit where the patient’s level of consciousness is not at issue, then this subtest can be omitted, since it does not contribute to any of the summary measures.

The first item asks whether the patient is sufficiently clinically stable to be approached. This determination should be made based on medical records or discussions with clinical staff. If the answer is ‘no’, then the evaluation does not proceed. The second item asks whether the patient can be roused. If the patient cannot be roused, then the evaluation does not proceed. In research studies of acute stroke or other patient groups where level of consciousness is potentially an issue, scores on these items can be used to systematically record unsuccessful attempts to evaluate.

The next three items probe orientation to place, time and person (e.g. *Can you tell me where we are right now?*). If the patient is unable to respond verbally, then three yes/no follow-up questions are asked. The next two items test whether the patient can follow simple commands: *Close your eyes* and *Squeeze my hand*. If either command is not followed, the examiner models the action. Alternative simple commands may be substituted if situational or patient factors dictate.

At this point, the examiner should be in a position to answer the question: *Can the patient stay awake*, *maintain attention*, *and attempt to follow commands?* If the answer is ‘yes’ or ‘yes, with reservations’ then the evaluation proceeds, otherwise it does not. This subjective question was chosen rather than defining a cutoff score based on the previous items, because of the many and various ways in which language deficits can impact the questions that probe orientation and ability to follow commands.

### Subtest 2: Connected speech

**Fig 3:**
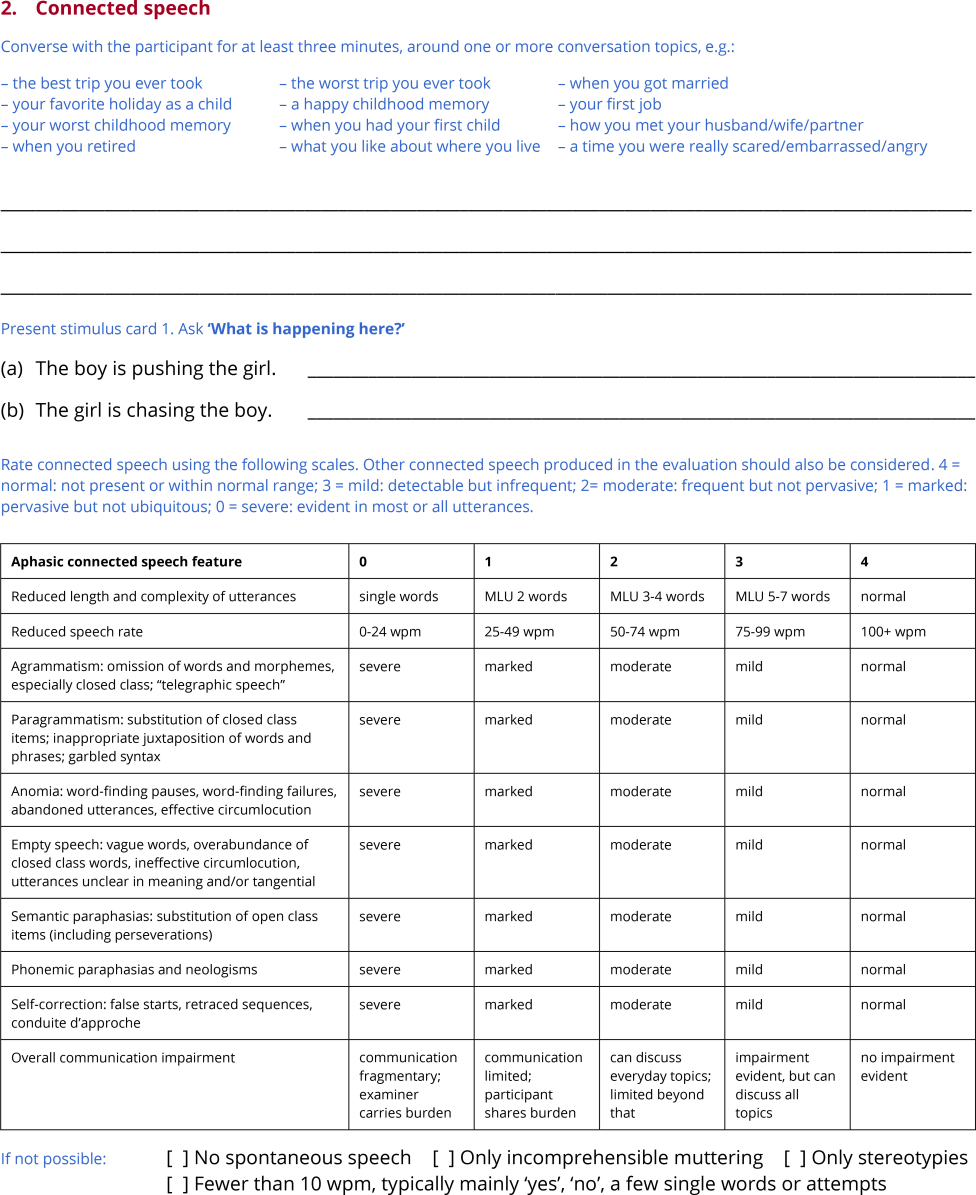
Subtest 2: Connected speech.

Connected speech provides valuable information in the assessment of aphasia, because it is quick and easy to obtain, while at the same time sensitive to underlying impairments in a wide range of language domains [8,35,36]. The examiner converses with the patient for at least three minutes, but ideally for five minutes. Any topic(s) of conversation can be used, and some examples are provided on the score-sheet: the best trip you ever took, when you get married, your first job, etc. (Fig 3). Additionally, two pictures of transitive actions are provided, which the patient is asked to describe in sentences (Fig 1A). Because pictures clearly define the target concept to be produced, they can sometimes be a helpful complement to open-ended conversation.

When it is impossible to obtain a connected speech sample, the examiner indicates why not, choosing from the following: ‘No spontaneous speech’; ‘Only incomprehensible muttering’; ‘Only stereotypies’; ‘Fewer than 10 wpm (typically mainly ‘yes’, ‘no’, a few single words or attempts)’. These are the four most common situations in which speech samples cannot be meaningfully analyzed [37].

Ten features of the connected speech sample are then rated qualitatively on a 5-point scale from 0 to 4. For most of the features, the scale points are defined as follows: 4 = normal: not present or within normal range; 3 = mild: detectable but infrequent; 2 = moderate: frequent but not pervasive; 1 = marked: pervasive but not ubiquitous; 0 = severe: evident in most or all utterances. This scale is based on a scale for rating motor speech features described by Strand and colleagues [38]. For a few of the features, this scale is not appropriate. For ‘Reduced length and complexity of utterances’, the scale points are defined in terms of approximate mean length of utterance, and for ‘Reduced words per minute’, the scale points are defined in terms of words per minute. In neither case is there an expectation that the examiner literally counts words or uses a stopwatch; a general impression is what is sought. Finally, for ‘Overall communication impairment’, the scale is based closely on the Aphasia Severity Rating Scale from the BDAE [17], though the most severe point from that scale is absent because it would correspond to situations where no connected speech sample can be obtained.

While scores of 4 (not present) are generally associated with intact function, this is not always the case. For example, a patient who produces only single-word utterances will not generally exhibit paragrammatism. Scores of 4 in such cases will not result in the over-estimation of the patient’s abilities, because of the way that summary measures are calculated (see below). Also note that 4 is defined as ‘not present or within normal range’. This is because unimpaired speakers occasionally have word finding difficulties, retrace their utterances, and so on. Therefore a patient who only occasionally struggles to find a word, or sometimes corrects his or her sentences, may receive scores of 4 on the relevant features if their difficulties are within the range of normal.

Finally, all connected speech that the patient produces during the administration of the QAB should be considered, not just the speech sample that is deliberately elicited. Therefore, it may be necessary to revise these ratings later as the evaluation proceeds.

### General administration and scoring principles for subtests 3 through 7

**Fig 4:**
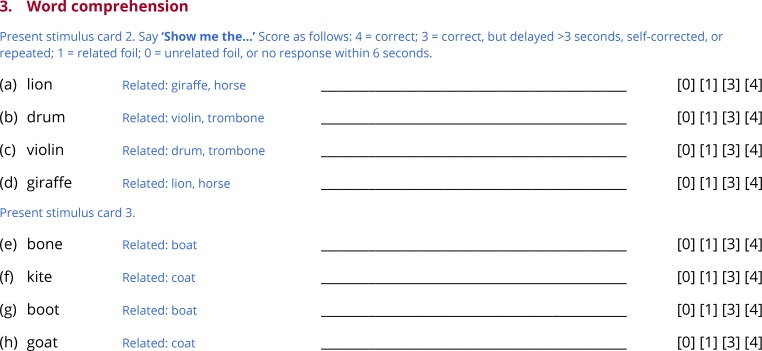
Subtest 3: Word comprehension.

**Fig 5:**
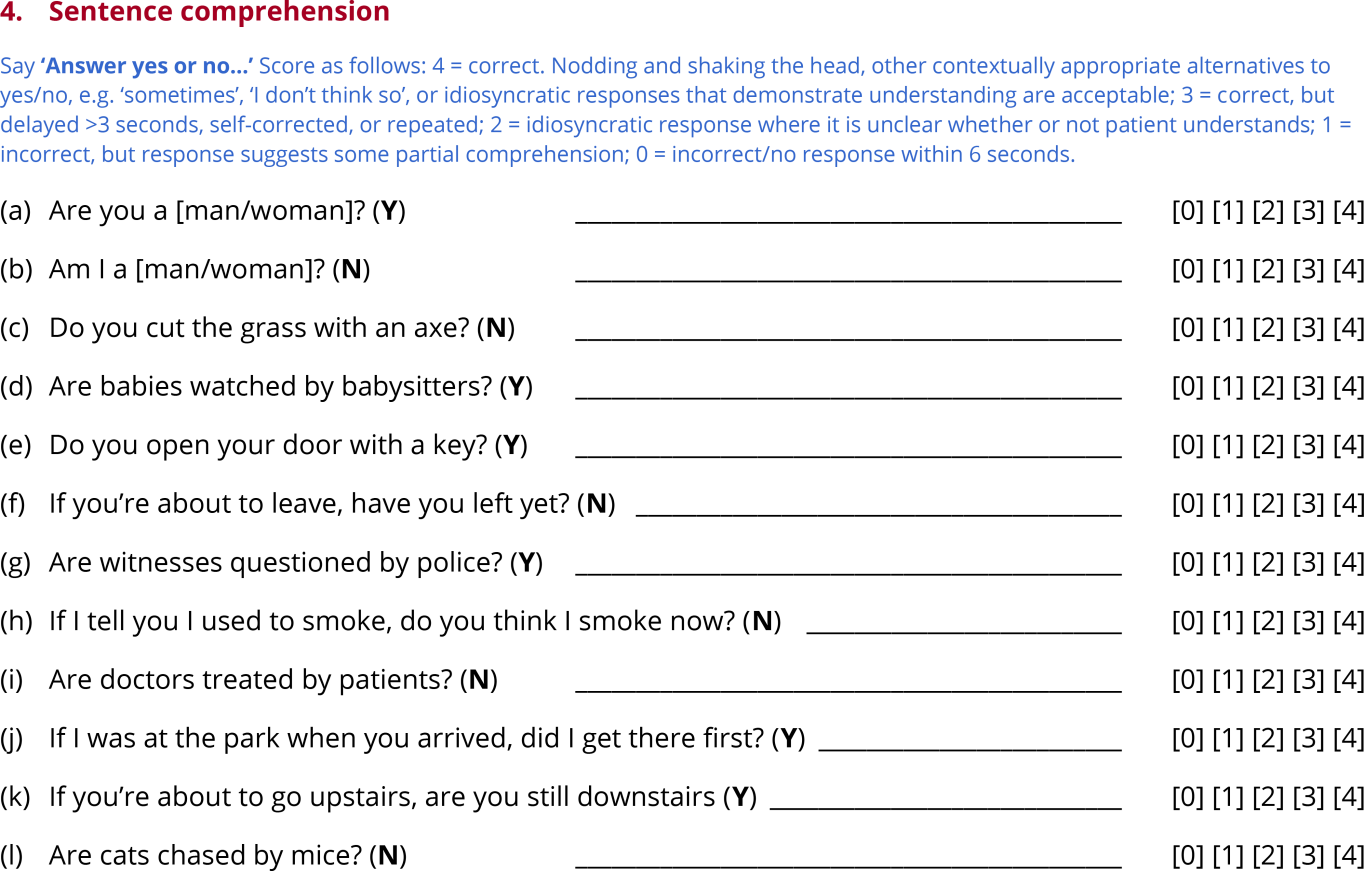
Subtest 4: Sentence comprehension.

**Fig 6:**
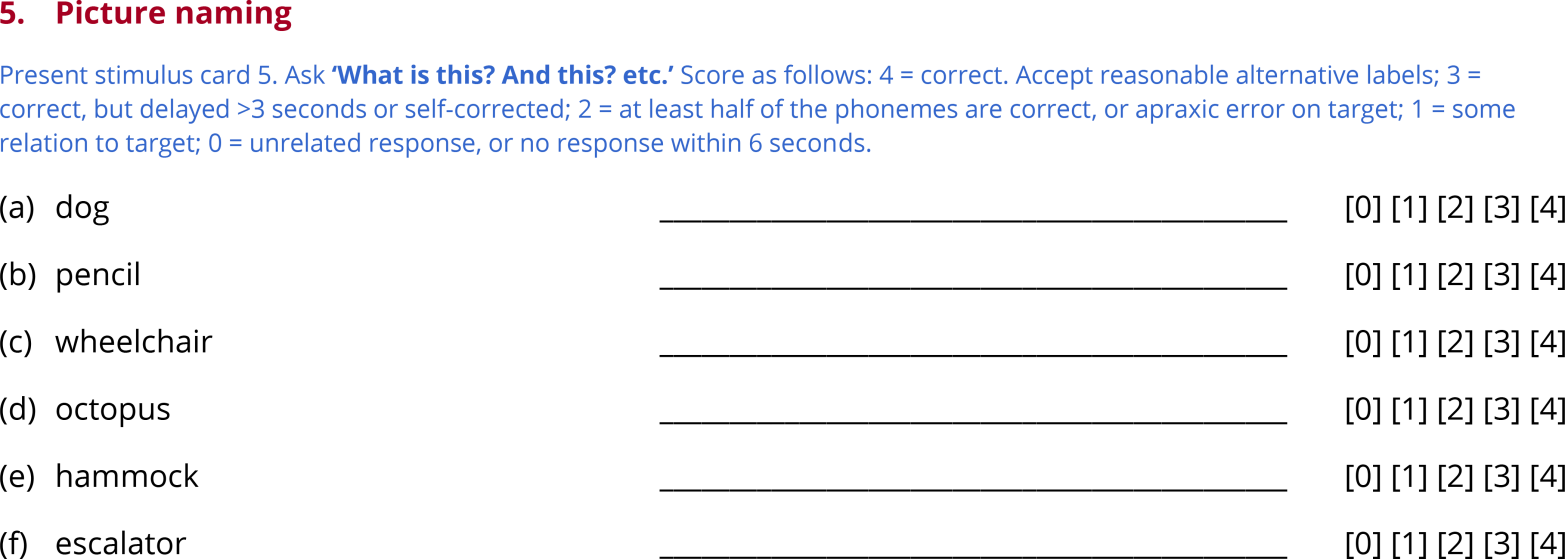
Subtest 5: Picture naming.

**Fig 7:**
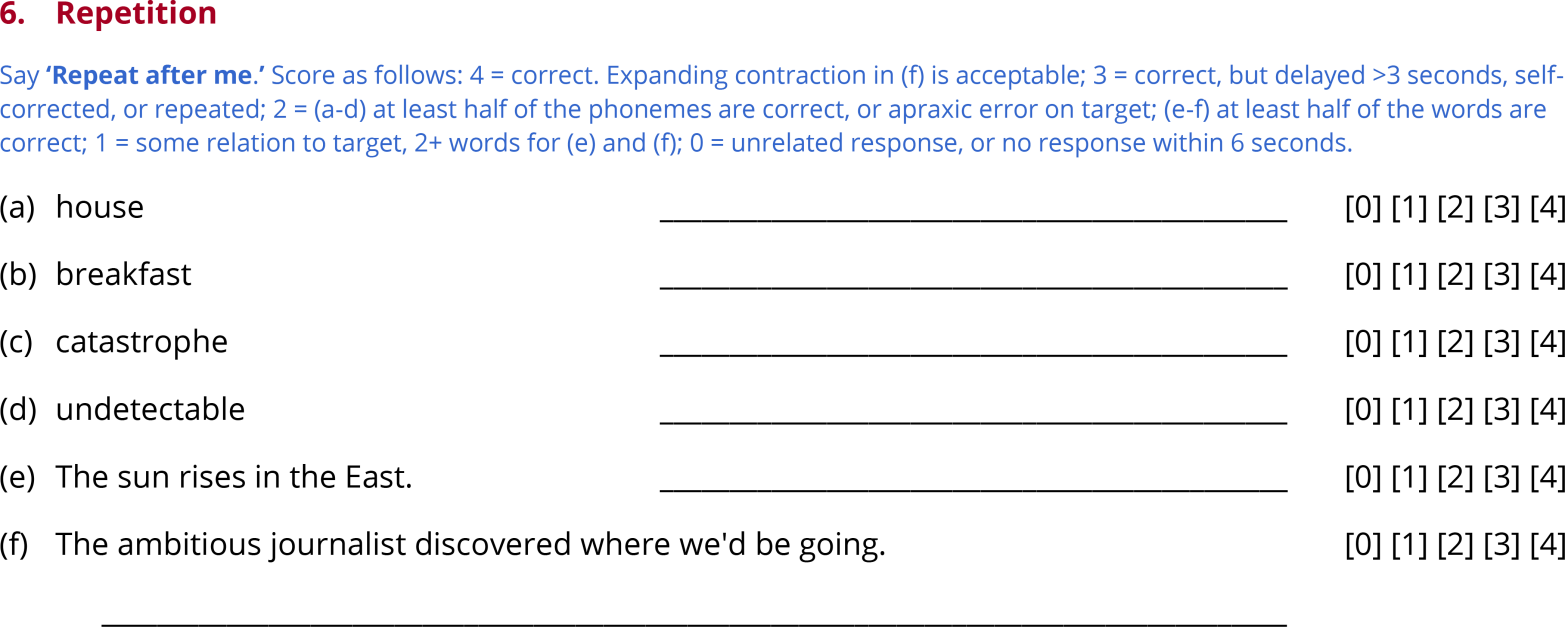
Subtest 6: Repetition.

**Fig 8:**
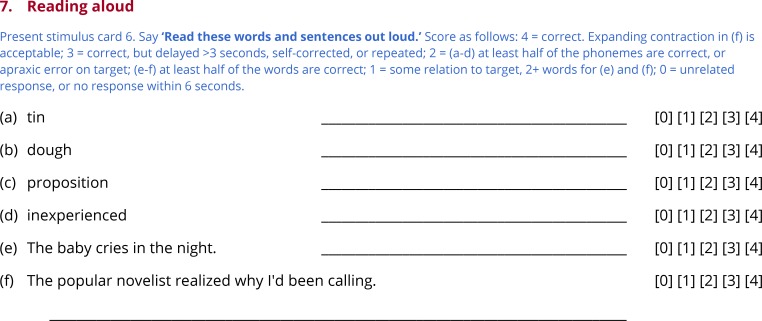
Subtest 7: Reading aloud.

Subtests 3 through 7 test comprehension of words (Fig 4) and sentences (Fig 5), picture naming (Fig 6), repetition (Fig 7) and reading (Fig 8). In this section, general principles are described for administering and scoring these subtests.

#### Timing of responses

To receive the maximum score of 4 on any item, a correct response must be initiated within 3 seconds. The response itself may extend beyond 3 seconds, and in some cases, it often will, such as items involving sentential responses. A correct response initiated between 3 and 6 seconds receives a score of 3. A score of 3 is also applied to sentence responses that are initiated within 3 seconds but contain within them a delay of 3 seconds or more. If no response is initiated within 6 seconds, the item scores 0 (no response). Even though correct or incorrect responses after 6 seconds could potentially be informative, a short response window was defined in the interest of efficiency; when a patient does not provide a response, the examiner only has to wait 6 seconds before moving on.

#### Multiple responses

Individuals with aphasia often make multiple responses to items. In the QAB, only the first complete response is scored. This was a choice made in the interest of efficiency and simplicity, as well as sensitivity, because errors, even if corrected, are informative as to the severity and nature of aphasia. A complete response is defined as a word or sentence that is an attempt at the item (i.e. not *um*, *uh*, *let me see*, etc.) and that is complete (i.e. not a false start, such as *k-* or *t*-, or an audibly abandoned fragment, e.g. *bala*-). If the patient produces a complete response, then subsequently self-corrects it to another response, only the first response is scored; any subsequent self-corrections are ignored for the purpose of scoring. However, if the patient produces one or more false starts or fragments, and then produces the correct response, a score of 3 is assigned. For sentence items, a score of 3 is also assigned if the sentence contains any false starts, fragments, or retracings (sequences of one or more complete words that are made redundant by subsequent repetitions, amendments, elaborations or alternative expressions).

#### Repeating items

Items that involve a verbal prompt from the examiner may be repeated once, at the patient’s request, within 6 seconds, but not if they have already attempted the target. If an item is repeated, restart the 6 second count. A correct answer after a request for repetition is scored 3. If an item needs to be repeated for situational reasons, such as a door slamming in the background, this does not count as a repetition for the purpose of scoring, and does not need to be noted. An item may also be repeated if the examiner suspects that the patient misheard the stimulus, even after a response has been provided. This also does not count as a repetition for the purpose of scoring. For interpersonal reasons, it may be desirable to provide requested repetitions even when they are not permitted according to the scoring system. This can be done, so long as any subsequent responses are ignored for scoring purposes.

#### Motor speech

All manifestations of dysarthria, as well as apraxic errors that do not affect the identity of any phoneme (e.g. elongation of a phoneme), are disregarded for scoring purposes. Apraxic errors that do affect the identity of one or more phonemes have an impact on scoring, as described below for Subtest 5. Motor speech deficits are assessed directly in Subtest 8.

### Subtest 3: Word comprehension

To test whether the patient can map word forms onto their referents, the examiner produces eight object names, and the patient is asked to identify each object in an array containing semantic or phonological foils (Fig 4). There are two pages of stimulus arrays. On the first, three of the objects are animals, and three are musical instruments (Fig 1B). The examiner says ‘Show me the…’ and names one of the objects (e.g. *lion*). In total, four of the six objects are named. The presence of multiple semantic foils should make these items challenging for patients with semantic deficits. The second page is laid out the same way, except that now there are phonological foils (Fig 1C): each of the four items that is named has at least one other object on the page whose name differs by a single phoneme (e.g. in the example provided, the objects named are *bone*, *kite*, *boot* and *goat*, and the foils are *boat* and *coat*). The presence of phonological foils should make these items challenging for patients with receptive phonological deficits.

The objects to be identified differ across the three forms of the QAB, but are matched based on lexical frequency. All of the pictures used in this subtest were taken from the stimulus set created by Rossion and Pourtois [39]. Item difficulty is not graded in this subtest, because word comprehension scores from graded tests involving difficult items are highly dependent on level of education [40], and so would be difficult to interpret. Just two panels of objects were used for the eight items to minimize the need for page turning and for the patient to have to visually process large numbers of objects. This approach has a slight disadvantage in that guessing becomes more effective on later trials, if objects already identified are ruled out. To partially mitigate this problem, only four of the six objects are named, which reduces the ability to guess on later trials.

### Subtest 4: Sentence comprehension

To probe sentence comprehension, the examiner says ‘Answer yes or no’, and then asks twelve questions, each of which can be answered based on situational or real world knowledge (Fig 5). For non-verbal patients, ‘thumbs up’ and ‘thumbs down’ icons are provided that can stand in for ‘yes’ and ‘no’ (Fig 1D).

The questions fall into four categories. First, there are two very simple situational questions, e.g. *Are you a man?, Am I a woman?* Second, there are two simple questions about typical instruments used to perform actions, e.g. *Do you cut the grass with an ax?, Do you open your door with a key?* Correctly answering these first two types of questions requires multiple lexical items to be considered in relation to one another, but does not depend on syntax.

The third type of questions are passive voice transitive sentences in which both of the noun phrases are plausible agents and patients for the verb, e.g. *Are doctors treated by patients?, Are babies watched by babysitters?* These questions depend on syntactic representations, because the agent and patient do not appear in their prototypical order, as signaled by the passive voice morphosyntax. Such sentences have been widely used in the literature on syntactic deficits in aphasia [13], usually along with active voice counterparts. Active voice counterparts were not included in the QAB because the goal was not to compare these two conditions, but rather to generate sentences that depend on access to syntactic representations.

The fourth type of sentences revolve around complex aspectual and modal distinctions, e.g. *If you’re about to leave*, *have you left yet?; If I tell you that I used to smoke, do you think I smoke now?* Answering these questions requires access to subtle grammatical knowledge about auxiliary and modal verb constructions. Aspectual and modal contrasts have not often been investigated in individuals with aphasia [41], but encoding aspectual and modal distinctions is a central function of morphosyntax, and moreover, the challenging nature of these sentences completes a continuum of difficulty across the twelve items.

All of the questions can be answered based on situational or real world knowledge. This contrasts with the more commonly used approach of testing sentence comprehension in aphasia: sentence-picture matching, for example in the CAT [19]. The disadvantage of sentence-picture matching is that patients have to process all the pictures (which generally involve the same entities interacting in different ways), and the sentence, and then find the picture that matches the sentence. This is heavily dependent on executive, attentional, and working memory resources, and is often surprisingly time-consuming, even in patients with intact syntactic comprehension. In contrast, with sentences relying on situational or real world knowledge, the non-linguistic demands of the task are considerably reduced, because the truth criterion against which the sentence is being evaluated is pre-existing, unlike a complex array of similar-looking pictures. One limitation of this approach is that it depends on world knowledge being intact. Fortunately, this is usually the case in most forms of aphasia [42], with the exception of semantic dementia, for which a sentence-picture matching task would be more appropriate [43]. Another limitation is that because there are only two possible responses for each item, more items are needed to reliably estimate performance.

Most of the items were constructed from scratch, although a few are based on other aphasia batteries. The items used in the three forms of the QAB are nearly identical in their syntactic structures, and all use relatively familiar vocabulary in order to minimize demands on comprehension of individual words.

### Subtest 5: Picture naming

Anomia is the most ubiquitous deficit in aphasia, and confrontation naming is a well-established means of quantifying its severity. The examiner presents the next stimulus card (Fig 1E), containing six pictured objects, points to the first, and asks ‘What is this?’. Each of the six objects is tested in turn (Fig 6).

Sixteen of the 18 items used across the three forms of the QAB come from the Boston Naming Test (BNT) [44]. Unlike the line drawings in the BNT, color pictures were used, either from Rossion and Pourtois [39] or downloaded using Google image search. Two very easy items were added—*dog* and *book*—to match *bed*, which is by far the easiest item of the BNT. The six items for each form were selected to span a wide difficulty range, based on the item response theory analysis of the BNT reported by del Toro and colleagues [45].

Some further explanation is required regarding scoring of partially correct responses. A score of 2 out of 4 is assigned for responses that are phonologically related to the target, when at least half of the phonemes are correct. To assess this, the number of substitutions, additions, deletions, or transpositions that would be necessary to transform the form produced into the target are counted. If this number is less than or equal to half of the number of phonemes in the target form, then the item should be scored 2. For instance, with the target *harmonica*, the real word response *harmony* would receive as score of 2, as would a nonword response such as [hɑɹnɑmɪkə]. Scores of 2 are also assigned to apraxic errors that are attempts at the correct target. Like phonological errors, apraxic errors may include substitutions, additions, deletions or metathesis of phonemes. However they also typically entail distorted phonemes and other apraxic features [38]. There is no need to count operations as for phonological errors, which would often be impossible given that apraxic errors often cannot be characterized in terms of a linear sequence of discrete phonemes. Rather, any apraxic error that is clearly an attempt at the target is scored 2. The only exception is for minor apraxic errors such as distortions or elongations that do not impact the identity of any phoneme, which are ignored for scoring purposes. Finally, words that are morphologically related to the target also score 2, e.g. *harmonicas*.

Scores of 1 are assigned to responses that bear some relation to the target. This may include semantically related words (e.g. *flute*), phonologically related words that are less than half correct (e.g. *harbor*), neologisms with some relation to the target (e.g. [hɑɹpɪlki]), and circumlocution (e.g. *you play it with your mouth*; *Bob Dylan*; *instrument*). Scores of 0 are assigned to unrelated words, neologisms with no relation to the target, and circumlocution with no relation to the target (e.g. *it’s some kind of a thing*).

### Subtest 6: Repetition

Repetition is an informative function to assess because the availability of the target form tends to highlight encoding processes, and also because relatively spared repetition is a hallmark of transcortical aphasias. The examiner states ‘Repeat after me’, then produces each item in turn (Fig 7).

The first item on each form is a high-frequency monosyllabic concrete noun with a CVC shape (no consonant clusters) (e.g. *house*). The second item is a medium-frequency disyllabic concrete noun with an initial two-consonant cluster (e.g. *breakfast*). The third item is a low-frequency four-syllable noun with at least one consonant cluster, and travel between multiple places of articulation (e.g. *catastrophe*). The fourth item is a low-frequency five-syllable morphologically complex adjective (e.g. *undetectable*). The fifth item is a simple 6-word sentence (e.g. *The sun rises in the East*). The sixth item is an 11-word sentence that contains several low- to medium-frequency lexical items, as well as a complex string of function words (e.g. *The ambitious journalist discovered where we’d be going*.). Taken together, the repetition items span a range of difficulty, and include items that pose challenges for speech motor programming, lexical access, phonological encoding, and morphosyntax.

The first four items, which are single words, are scored the same way as the picture naming items. The fifth and sixth items, which are sentences, are scored similarly, except that scores of 2 are defined differently. Instead of counting phonemes, the number of substitutions, additions, deletions, or transpositions of words that would be necessary to transform the sentence produced into the target sentence are counted. If this number is less than or equal to half of the number of words in the target form, the item is scored 2.

### Subtest 7: Reading aloud

To minimize administration time, the QAB assesses only one aspect of reading: the ability to read aloud (Fig 8). Reading aloud requires mapping of orthographic word forms to phonological word forms, either through productive sublexical processes or through stored associations. The examiner states ‘Read these words and sentences out loud’ and, if necessary, uncovers the six items one at a time (Fig 1F).

The items for reading aloud are structured the same as the repetition items except in one respect: the second item for reading is a low-frequency concrete noun with an atypical spelling-sound correspondence (e.g. *dough*). The reading items are scored in the same way as the repetition items.

### Subtest 8: Motor speech

**Fig 9:**
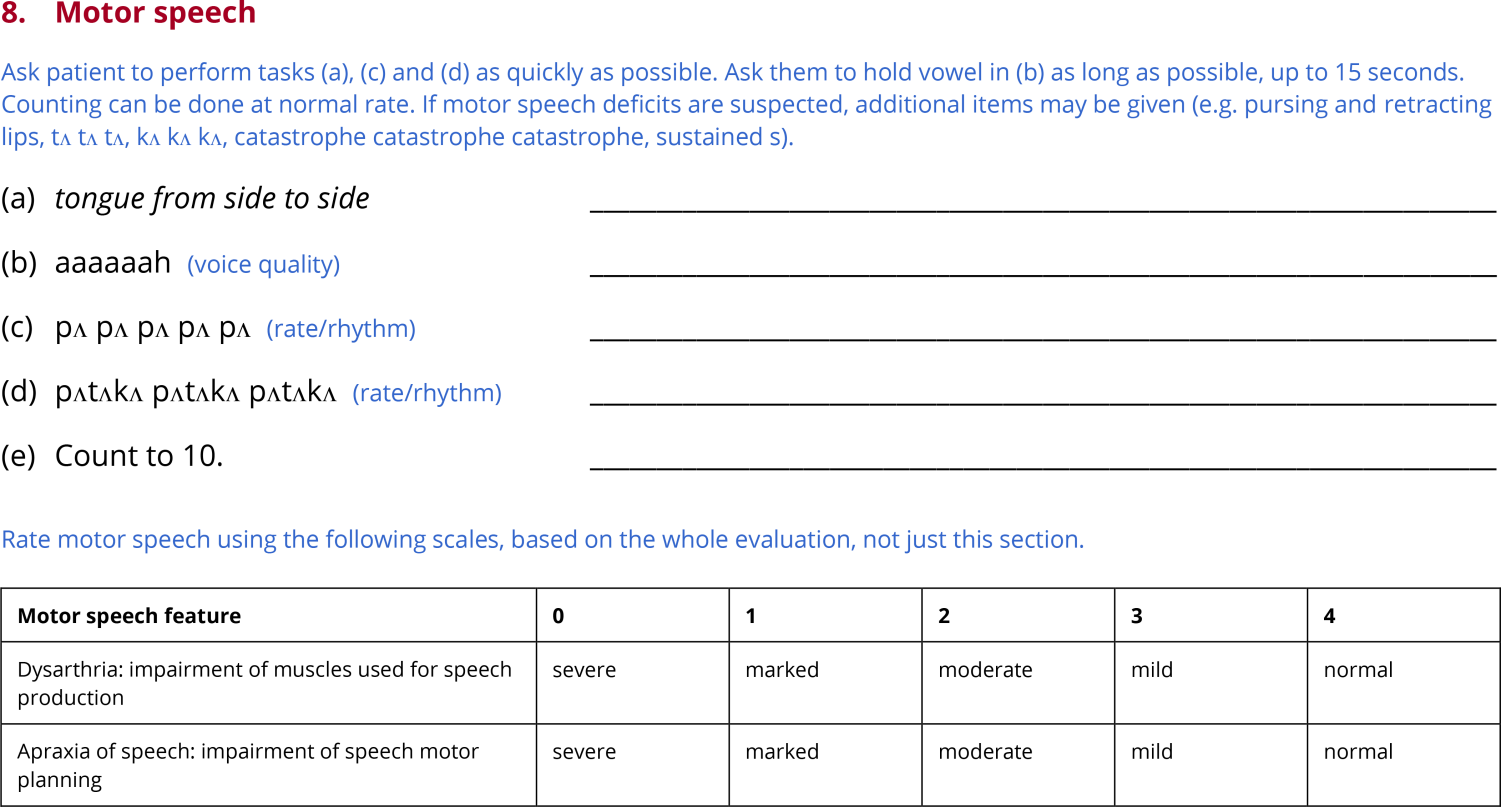
Subtest 8: Motor speech.

Many individuals with aphasia, especially non-fluent forms, present with concomitant apraxia of speech. Some patients, especially stroke patients in the acute to subacute stage, may present with dysarthria, usually unilateral upper motor neuron dysarthria. Apraxia of speech, and the form of dysarthria most often associated with aphasia, can be identified and quantified quite effectively based on the speech production components of any aphasia battery, along with examination of alternating motion rate (AMR) and sequential motion rate (SMR) (Strand et al., 2014). Therefore the final subtest of the QAB is a brief motor speech evaluation which includes AMR and SMR tasks, along with a few other basic motor tasks (Fig 9).

The examiner asks the patient to move their tongue from side to side as rapidly as possible, testing oro-motor function. Then, the patient is asked to take a deep breath and hold the vowel [*a*] for as long as possible, probing respiratory support and voice quality. The patient is then asked to rapidly repeat the syllable [pʌ] (AMR) and the sequence [pʌtʌkʌ] (SMR); differential impairment of the latter is characteristic of apraxia of speech. Finally the patient is asked to count to ten (automatic speech), which is useful for checking for any motor impairment in the absence of a significant planning component. Additional assessments can be added on an ad hoc basis if required (e.g. repeating [tʌ] and [kʌ], repeating *buttercup* instead of [pʌtʌkʌ], etc.)

Based on the motor speech subtest as well as the rest of the QAB, apraxia of speech and dysarthria are scored on the same 5-point scale used for most connected speech features [38,46].

### Calculation of summary measures

From the eight subtests of the QAB, eight summary measures are derived: (1) Word comprehension; (2) Sentence comprehension; (3) Word finding; (4) Grammatical construction; (5) Speech motor programming; (6) Repetition; (7) Reading; and (8) QAB overall. Each summary measure ranges from 0 (impaired) to 10 (unimpaired). Table 1 shows how each summary measure is calculated. A Macro is provided to calculate summary measures (S1 Macro).

**Table 1:** Calculation of summary measures.

Summary measure	Definition	
Word comprehension	Word comprehension total, corrected for chance by subtracting 8 and clipping at 0; denominator is now 24	
Sentence comprehension	Sentence comprehension total, corrected for chance by subtracting 24 and clipping at 0; denominator is now 24	
Word finding	60%	Picture naming total
	20%	Connected speech: Anomia
	20%	Average of Connected speech: Empty speech, Semantic paraphasias, and Phonemic paraphasias, but capped so as not to exceed Anomia
Grammatical construction	40%	Connected speech: Agrammatism
	20%	Connected speech: Reduced length and complexity
	20%	Connected speech: Paragrammatism, but capped so as not to exceed Agrammatism
	20%	Average of sentence items from repetition and reading subtests
Speech motor programming	Motor speech: Apraxia of speech	
Repetition	Repetition total	
Reading	Reading aloud total	
QAB overall	18%	Word comprehension summary measure
	18%	Sentence comprehension summary measure
	14%	Word finding summary measure
	14%	Grammatical construction summary measure
	8%	Speech motor programming summary measure
	8%	Repetition summary measure
	8%	Reading summary measure
	8%	Connected speech: Overall communication impairment
	2%	Connected speech: Reduced words per minute
	2%	Connected speech: Self-correction

All summary measures are out of 10, and are calculated by dividing the score described by its denominator, then multiplying by 10, or the appropriate percentage of 10 as indicated.

On the word comprehension and sentence comprehension subtests, correct responses will sometimes reflect guessing. Therefore the expected total that would result from guessing is subtracted from the actual total, prior to rescaling onto the (0, 10) scale.

Word finding is composed of the picture naming score (60%), and of connected speech scores related to word finding, which are more ecologically valid, though harder to quantify (40%). The anomia score from the connected speech sample measures word finding most directly, so it is weighted 20%. The scores for semantic paraphasias, phonemic paraphasias and empty speech together make up the other 20%, however for the purpose of calculating this summary measure, these scores are capped so as not to exceed the anomia score. This is to avoid overestimating the word finding abilities of patients who make few errors only because they produce few words.

Eighty percent of the grammatical construction measure is derived from connected speech measures. Agrammatism contributes 40% of the score, and reduced length and complexity of utterances contributes another 20%. Paragrammatism contributes 20%, but is capped so as not to exceed the agrammatism score. This is to avoid overestimating the grammatical function of patients whose limited production of grammatical words and morphemes excludes the possibility of exhibiting features of paragrammatism. The remaining 20% of the grammatical construction summary measure reflects scores on the sentence items from the repetition and reading subtests.

The speech motor programming summary measure is derived simply by rescaling the apraxia of speech score from the motor speech subtest. Similarly, the repetition and reading summary measures are derived simply by rescaling the total scores of the repetition and reading subtests.

The QAB overall score is derived from the seven other summary measures, along with three connected speech items that do not contribute to any of the other summary measures. The contributions of the seven summary measures are not equal. In particular, receptive measures are weighted more highly in order to yield a QAB overall measure that is more closely balanced between expressive and receptive deficits. It is also worth noting that speech motor programming (i.e. apraxia of speech) does contribute to the overall score. This reflects a view that although apraxia of speech and aphasia can dissociate, apraxic deficits are very common in non-fluent patients in particular, reflect left hemisphere damage, and interact closely with other components of fluency such as grammatically construction and word finding to yield an overall impression of non-fluency. Moreover, apraxic errors already will impact scores on other subtests such as picture naming, repetition, and reading. Therefore it seems more parsimonious to include apraxia of speech in the overall summary measure.

Regarding the three connected speech items that contribute to the QAB overall summary measure, overall communication impairment is weighted quite highly given its ability to capture a gestalt impression that goes beyond individual factors. Reduced words per minute and self-correction are taken into account because even though they are non-specific in the sense that they can potentially reflect deficits in several different language domains, these can be sensitive measures in the mildest patients.

### Participants

The QAB was administered to four groups of participants: acute stroke patients with aphasia, acute stroke patients without aphasia, chronic stroke patients with aphasia, and healthy controls (Table 2). All participants were native speakers of English, except for two of the individuals with chronic post-stroke aphasia and two of the healthy controls; these four participants were native speakers of Spanish whose primary language was now English and who were fluent in English.

**Table 2:** Demographic characteristics of the participants.

	Ac+A	Ac	Ch+A	HC
Number of participants	28	25	16	14
Age (years)	63.5 ± 17.3	59.6 ± 18.1	60.4 ± 14.8	53.1 ± 15.1
Sex (M/F)	14/14	11/14	12/4	8/6
Handedness (R/L)	25/2; 1 ambi	23/2	14/2	10/4
Education (years)	12.8 ± 2.8	13.2 ± 2.4	15.1 ± 2.5	17.1 ± 1.9
Race	27 W; 1 AA	22 W; 3 AA	13 W; 1 AA; 1 AIAN; 1 >1	12 W; 1 AA; 1 >1
Days post stroke	3.6 ± 1.5	2.6 ± 1.7	1955 ± 1220	N/A
Evaluation duration (minutes)	18.9 ± 7.3	11.6 ± 3.0	16.7 ± 6.0	Not timed

Ac+A = acute stroke patients with aphasia; Ac = acute stroke patients without aphasia; Ch+A = chronic stroke patients with aphasia; HC = healthy control participants; W = White; AA = African American; AIAN = American Indian or Alaska Native; >1 = More than once race

The study was approved by the Human Research Protection Program at Vanderbilt University and the Human Subjects Protection Program at the University of Arizona. All participants gave written informed consent, except in the case of patients whose comprehension deficits precluded a meaningful consent process; in these cases, written informed consent was obtained from a legally authorized surrogate.

#### Acute stroke patients

All patients who were seen by the stroke service at Vanderbilt University Medical Center in Nashville, Tennessee were screened for eligibility. The inclusion criteria were as follows: (1) acute stroke confirmed on CT or diffusion weighted MRI; (2) stroke localized wholly or predominantly to left hemisphere supratentorial regions (i.e. cortex, cortical white matter, basal ganglia, thalamus); (3) stroke extent at least 500 mm^3^; (4) aged 18 to 90; (5) fluent and literate in English premorbidly; (6) sufficiently medically stable to complete the QAB within 7 days of the stroke. The exclusion criteria were as follows: (1) dementia, or impaired cognitive or language function at baseline for any other reason; (2) major psychiatric disorders; (3) serious substance abuse or withdrawal. Note that previous stroke was not an exclusionary criterion, so long as there were no cognitive or language deficits that persisted at the time of the new stroke.

Over a 7½-month period, 98 patients met all criteria. Of those, 71 were approached to request written informed consent. 58 patients consented to participate and were administered the QAB, while 13 declined to participate. The remaining 27 patients were either not approached at all for situational reasons (e.g. being discharged prior to being approached), or were approached initially but not consented for situational reasons (e.g. medical procedures taking place).

The 58 patients who completed the QAB were divided into three groups based on clinical impression, not on performance on the QAB: (1) 28 patients with aphasia; (2) 25 patients with no aphasia, and no more than moderate dysarthria; (3) 5 patients who did not appear to have aphasia, but where diagnosis was compromised by marked or severe dysarthria. The first two of these groups were included in the study. The third group was not included, but their performance on the QAB will be briefly noted below.

#### Chronic stroke patients

Sixteen patients with chronic post-stroke aphasia were recruited from an aphasia center in Tucson, Arizona, or were prior participants in aphasia research at the University of Arizona. The inclusion criteria were (1) persistent and stable aphasia of any etiology; (2) aged 18 to 90; (3) fluent and literate in English premorbidly. The exclusion criteria were the same as described above for acute stroke patients. Of the 16 patients recruited, 15 had experienced left hemisphere strokes, and 1 had experienced bilateral strokes, with the right hemisphere stroke being more extensive.

#### Healthy controls

Fourteen healthy control participants were recruited mostly from a neighborhood listserv in Tucson, Arizona. They reported no neurological or psychiatric history. The Mini Mental State Examination was administered to each participant, and scores ranged from 27 to 30.

### Administration and scoring of reference data

The QAB was administered to the acute stroke patients with and without aphasia by SMS, a speech-language pathologist in her clinical fellowship year. These evaluations were all completed at patients’ bedsides at Vanderbilt University Medical Center. The mean administration time was 18.9 ± 7.3 minutes in individuals with aphasia, and 11.6 ± 3.0 minutes in individuals without aphasia (Table 2). There was no attempt to administer the QAB especially quickly, so administration time could readily be reduced further.

The 16 individuals with chronic post-stroke aphasia were tested 3 times each. Of these 48 evaluations, 38 were carried out by DKE, a licensed speech-language pathologist, 1 by SMW, a researcher, and 9 by students who were trained and supervised by DKE. Of the 14 healthy controls, 2 were tested by DKE and 12 by students supervised by DKE. All of these evaluations took place in quiet testing rooms at the University of Arizona. The mean administration time was 16.7 ± 6.0 minutes, again reflecting unhurried administration. During the first of the three sessions, the Western Aphasia Battery (WAB) was also administered and scored by DKE. The mean number of days between successive evaluations was 13.2 ± 9.7 (range 4 to 47).

All QAB evaluations were recorded with a Marantz PMD661mkII solid state recorder and a Sanken lavalier microphone (COS-11D), and videotaped with a GoPro Hero3+ (acute patients) or Canon Vixia HF S20 (chronic patients and healthy controls) for offline transcription and scoring. All evaluations were scored by SMS, and the 48 evaluations of patients with chronic aphasia were scored independently by JML, a speech-language pathologist in her clinical fellowship year. Both SMS and JML were trained by SMW in scoring procedures using data from other patients not included in the study.

Responses to each item were transcribed in full, except for connected speech samples, for which a sample of utterances were transcribed. Transcription and scoring was carried out using a custom database and web interface based on *postgresql*, *python*, *django* and *apache*. Scores were validated and summary measures were calculated using a custom MATLAB program (Mathworks, Natick, MA). Data analysis was carried out in MATLAB. In particular, inter-rater and test-retest reliability were assessed using intraclass correlation coefficients (ICC) [47].

## Results and discussion

Transcriptions and scores for each item for each participant are provided in S1 Dataset.

### Distributions of summary measures

**Fig 10:**
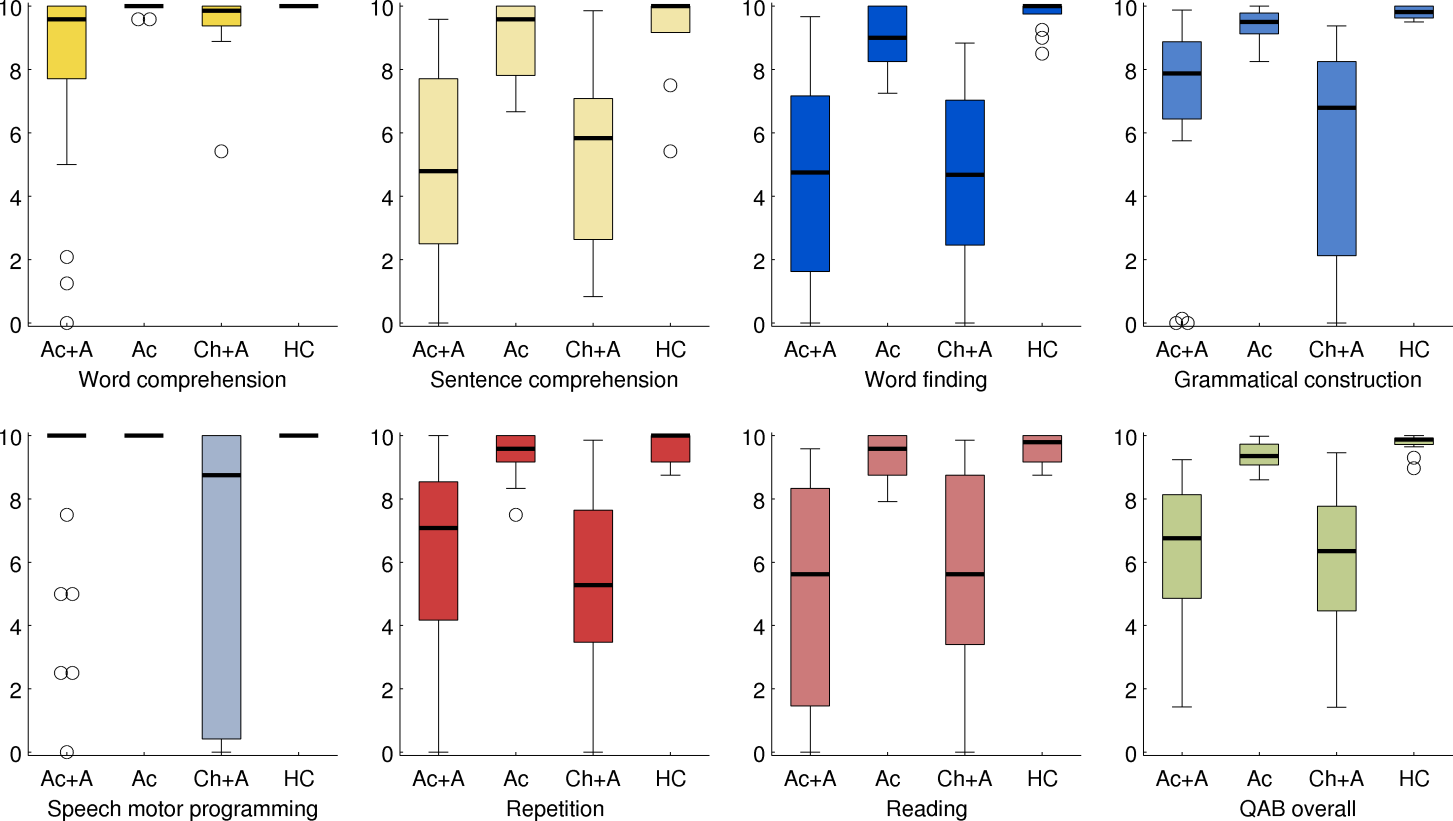
Distributions of the eight QAB summary measures. Boxes = interquartile range; whiskers = range not including outliers; plusses = outliers; thick horizontal lines = medians; Ac+A = acute stroke patients with aphasia (*n* = 28); Ac = acute stroke patients without aphasia (*n* = 25); Ch+A = chronic stroke patients with aphasia (*n* = 16); HC = healthy control participants (*n* = 14). Each measure is color-coded to match subsequent figures.

The distributions of the eight summary measures in the four groups of participants are presented in Fig 10. All eight measures showed wide distributions in the two groups of individuals with aphasia, which was expected since both groups were diverse in terms of aphasia severity and type. In the two groups of individuals without aphasia, all eight measures showed narrower distributions that were at or close to ceiling.

One notable limitation was observed, which was that there were apparent ceiling effects for the word comprehension measure. This was especially apparent in the individuals with chronic aphasia, only one of whom showed substantial impairment on this measure. Left hemisphere lesions often have only a modest effect on single word comprehension [40,48], which may reflect in part the capacity of the right hemisphere to comprehend single words [49]. While more difficult items could potentially have revealed milder single word comprehension deficits, the use of words that may not be familiar to all participants would run the risk of identifying deficits where none exist.

### Inter-rater reliability

**Fig 11:**
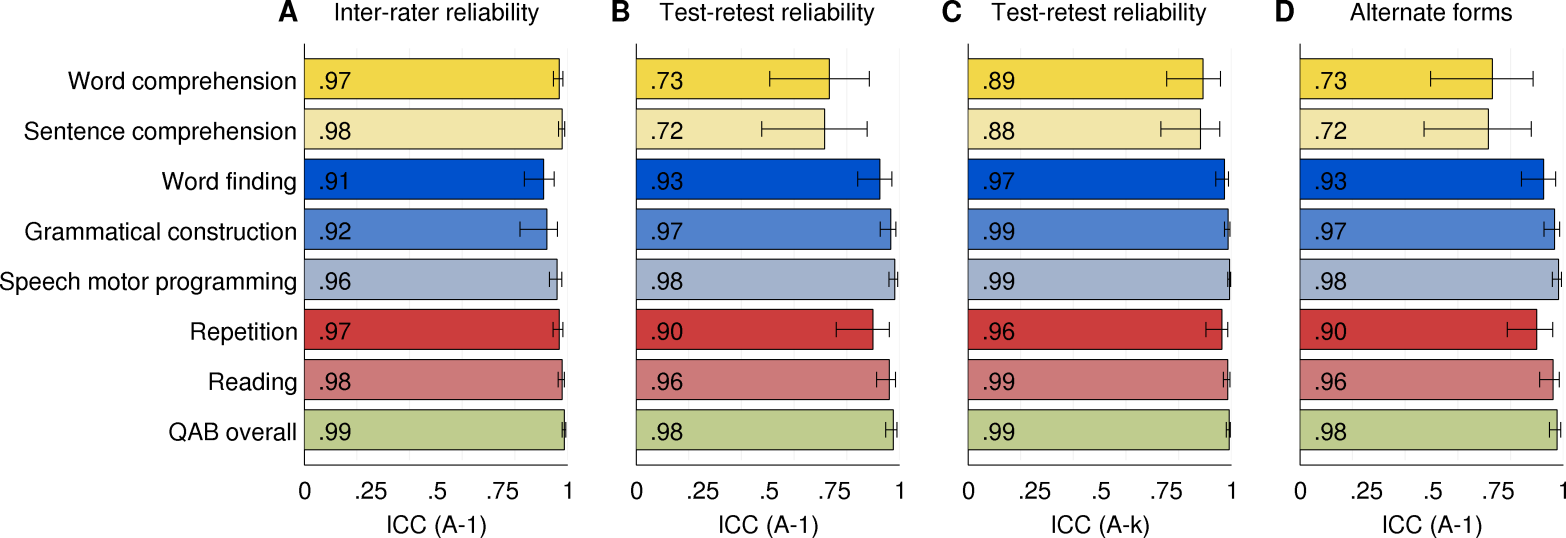
Inter-rater and test-retest reliability. (A) Inter-rater reliability of each summary measure, across two trained speech-language pathologists who each rated the same 48 evaluations from 16 individuals with chronic post-stroke aphasia. ICC type A-1 = intraclass correlation coefficient, absolute agreement. (B) Test-retest reliability of each measure, based on 16 individuals with chronic post-stroke aphasia who were each evaluated three times. ICC type A-1 = intraclass correlation coefficient, absolute agreement, correlation between any random pair of evaluations. (C) Test-retest reliability of each measure, based on 16 individuals with chronic aphasia who were each evaluated three times. ICC type A-k = intraclass correlation coefficient, absolute agreement, correlation between the means of sets of three random evaluations. Error bars denote 95% confidence intervals. (D) Alternate forms reliability (ICC type A-1).

Inter-rater reliability was calculated based on the 48 evaluations of the individuals with chronic aphasia, each of which was scored independently by two raters. The ICCs (type A-1) for each of the nine summary variables are shown in Fig 11A. The ICCs ranged from 0.91 (word finding) to 0.99 (QAB overall). These ICC values indicate excellent inter-rater reliability for all measures, according to the criteria defined by Cicchetti [50].

Note that these estimates of inter-rater reliability are based on scores from two speech-language pathologists who were carefully trained in the scoring of the battery, and scored it offline based on audiovisual recordings. Reliability of scoring in other circumstances will reflect the experience and training of the individuals who carry out the scoring.

### Test-retest reliability

Test-retest reliability was calculated based on the 16 individuals with chronic aphasia who were evaluated three times each on three separate occasions. Each summary measure was averaged across the two raters. The ICCs (type A-1) for each of the eight summary measures are presented in Fig 11B. Six of the measures had excellent test-retest reliability, ranging from 0.90 (repetition) to 0.98 (speech motor programming and QAB overall). The reliability of the two comprehension measures was good, not excellent: 0.73 for word comprehension and 0.72 for sentence comprehension. These somewhat lower ICCs reflect the limitation of small numbers of items in forced choice contexts.

Type A-k ICCs for test-retest reliability are shown in Fig 11C. These indicate the expected reliability if scores were averaged across all three forms of the QAB, i.e. three times as many items. All ICCs would have excellent reliability in this case, ranging from 0.88 (sentence comprehension) to 0.99 (four different measures). This shows that test-retest reliability can be markedly improved by administering multiple forms of the QAB and averaging scores across forms, if time permits.

None of the eight summary measures showed any learning effects, i.e. there were no measures that significantly increased over the three sessions (Session 3 > Session 1, all *p* > 0.05). In the 16 individuals with chronic post-stroke aphasia who completed all three forms of the QAB, there were no differences between forms on any of the eight summary measures (Repeated measures ANOVAs with Greenhouse-Geisser correction, all *p* > 0.05). Type A-1 ICCs for alternate forms reliability are shown in Fig 11D, and were almost identical to those for test-retest reliability.

### Sensitivity and specificity for presence versus absence of aphasia

**Fig 12:**
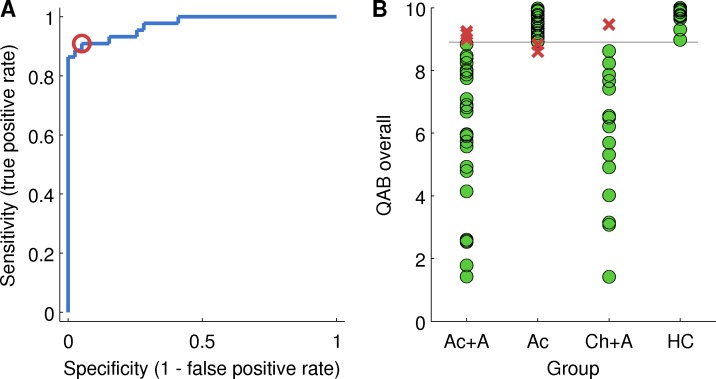
Determining a cutoff for diagnosis of aphasia. (A) Receiver operating characteristic (ROC) plot of sensitivity versus specificity. The sensitivity and specificity at the chosen cutoff of 8.9 is indicated with a red circle. (B) Diagnosis based on this cutoff, relative to clinical impression. Green circles = correctly diagnosed patients; Red crosses = incorrectly diagnosed patients.

The QAB overall summary measure was assessed as a metric for determining the presence or absence of aphasia. A receiver operating characteristic (ROC) curve is plotted in Fig 12A, showing the trade-off between sensitivity and specificity depending on the cutoff score for diagnosis of aphasia.

Based on the ROC curve, a QAB overall score of less than 8.90 was defined as indicative of aphasia. With this cutoff, the sensitivity of the QAB was 0.91 and the specificity was 0.95. In other words, 91% of patients with aphasia per clinical impression had a QAB overall score below 8.90, and of all the people with a score below 8.90, 95% had aphasia per clinical impression. This is illustrated in Fig 12B. Six individuals were misclassified: three patients with aphasia after acute stroke and one patient with chronic post-stroke aphasia scored above 8.90, while two patients with acute stroke but no aphasia scored below 8.90.

The misdiagnosed patient with chronic post-stroke aphasia was 8 years post stroke and had almost completely recovered; her WAB AQ was 98.8, so she did not meet WAB criteria for aphasia either. Still, her faint residual deficits were clinically appreciable in extended interaction, so she was retained in the sample. The three misdiagnosed acute patients with aphasia also had very mild aphasias that were nevertheless clinically appreciable.

The two misdiagnosed acute patients without aphasia made several errors on the QAB, especially in sentence comprehension, on which their scores were 6.7 and 7.9, however our clinical impression was that the mistakes they made reflected the impact of stroke on executive, attentional, and working memory processes, rather than aphasia.

As mentioned above, there were 5 acute stroke patients who did not appear to have aphasia, but where diagnosis was compromised by marked or severe dysarthria. These patients were not included in the study. However, it is noted that their overall QAB scores were 8.12, 8.16, 8.33, 8.46 and 9.03, i.e. four out of five scored below the cutoff score for aphasia. This indicates that scores in this range cannot be taken as indicative of aphasia when marked or severe dysarthria is present.

### Concurrent validity with respect to the Western aphasia battery

For five QAB summary measures, very similar measures can be derived from the WAB: QAB word comprehension corresponds to WAB auditory word recognition; QAB sentence comprehension corresponds to WAB yes/no questions and/or sequential commands; QAB word finding corresponds to the WAB naming subscore; QAB repetition corresponds to WAB repetition; and QAB overall corresponds to the WAB Aphasia Quotient. Another two QAB summary measures—grammatical construction, speech motor programming—correspond to some extent to the WAB fluency measure, which incorporates these two aspects of expressive language, among others. QAB reading corresponds to reading items in the supplementary parts of the WAB, which we did not administer.

**Fig 13:**
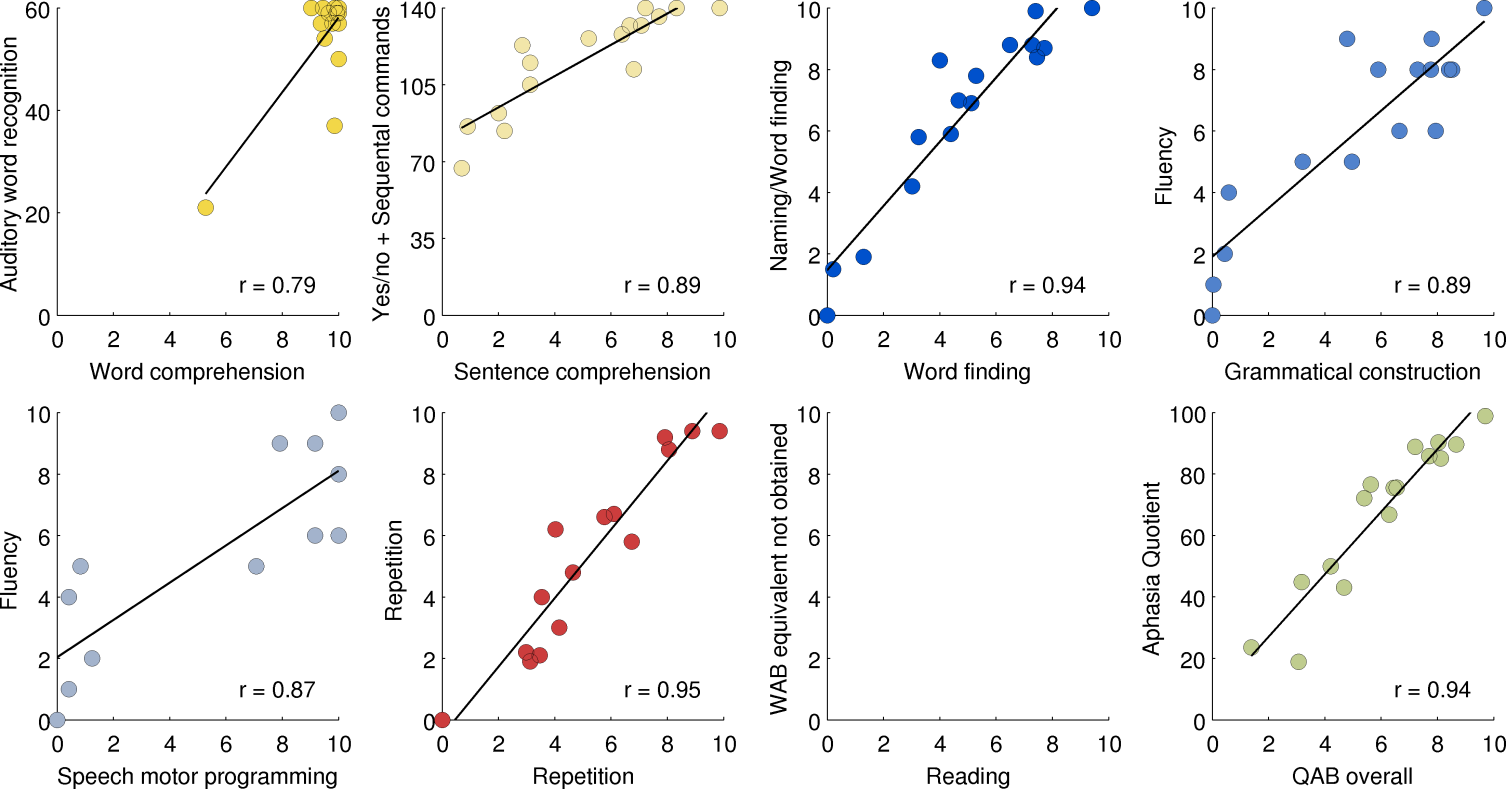
Concurrent validity with respect to the Western Aphasia Battery (WAB). Each QAB summary measure was correlated with most similar WAB measure, except for Reading, which was omitted because the written language section of the WAB was not administered. All correlations were significant (p < 0.001). Aud word rec = Auditory word recognition; Yes/no+SC = the sum of Yes/no questions and Sequential commands; Naming/WF = Naming and word finding; AQ = Aphasia quotient.

For the five measures with direct counterparts, and the two measures related to Fluency, correlations were calculated between each QAB measure (averaged across the three sessions and the two raters) and the relevant WAB measure (Fig 13). All measures were highly correlated with their WAB counterparts, with *r* ranging from 0.79 (QAB word comprehension with WAB auditory word recognition) to 0.95 (QAB repetition with WAB repetition) (*p* < 0.001 for all). Possible ceiling effects were apparent for the WAB measures of sentence comprehension and naming.

The weakest correlation, between the single word comprehension measures, was driven by a single outlier who was identified as impaired in this domain by both batteries. It is noteworthy that two other patients performed markedly worse on WAB auditory word recognition than they did on QAB word comprehension, due mainly to errors on body parts and left-right confusion, which are tested on the WAB but not the QAB.

Both grammatical construction and speech motor programming were highly correlated with the WAB fluency subscore, which conflates these constructs (*r* = 0.89 for grammatical construction; *r* = 0.87 for speech motor programming). Grammatical construction and speech motor programming were themselves highly correlated in these 16 patients (*r* = 0.95).

### Patient profiles

**Fig 14:**
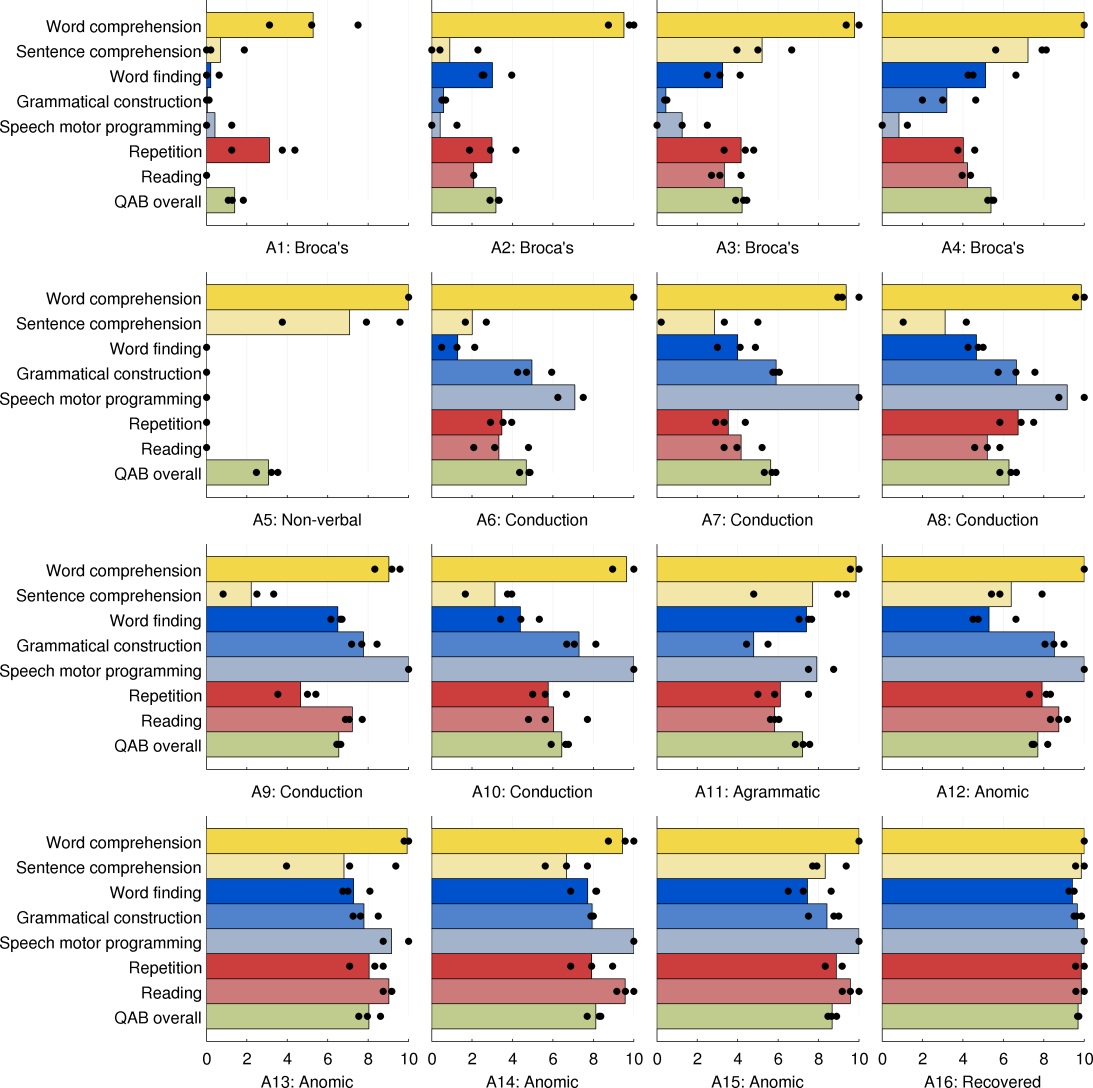
Profiles of QAB summary measures for each of the 16 individuals with chronic aphasia. The patients are arranged in groups according to clinical impression, then in ascending order of overall QAB score within each group. Patient scores were averaged across three sessions and two raters, but the scores for each of the three sessions are indicated with black circles to give a sense of test-retest reliability.

The QAB aims not only to determine the presence or absence of aphasia and quantify its severity, but also to quantify relative impairment or sparing in multiple language domains. Accordingly, the profiles of the 16 chronic post-stroke patients were examined, to determine whether the QAB is sensitive to different patterns of deficits in different aphasia types (Fig 14).

Four patients (A1, A2, A3, A4) presented with Broca’s aphasia per clinical impression, spanning a continuum of severity. Word comprehension was well preserved except in the first, most severe, patient. The most severely impaired domains were speech motor programming and grammatical construction, while sentence comprehension, word finding, repetition and reading were variably impaired according to the degree of overall severity. It is noteworthy that patient A4 met WAB criteria for conduction aphasia, not Broca’s aphasia, due to a Fluency score of 5, which was confirmed independently by a second rater. However his prominent agrammatism, halting, effortful speech, and apraxia of speech left us in little doubt that Broca’s aphasia was the most fitting clinical diagnosis. The QAB appropriately showed him to have a similar profile to the other patients with Broca’s aphasia, albeit less severe.

The next patient, A5, was completely non-verbal, and met WAB criteria for Broca’s aphasia. However he was very different to A1, the other patient with a similarly low AQ and QAB overall score. Unlike A1, his single word comprehension was intact, and his sentence comprehension was also quite good, comparable to A4, the least impaired individual with Broca’s aphasia. The QAB proved to adequately capture these important differences between these two patients.

Five patients (A6, A7, A8, A9, A10) presented with conduction aphasia per clinical impression, and all met WAB criteria for conduction aphasia. They spanned a narrow spectrum of severity. Word comprehension was uniformly good and sentence comprehension was uniformly impaired, as might be expected in patients with posterior temporal damage [48,51], which was the case in all five of these patients. Word finding, grammatical construction, repetition and reading were generally moderately but variably impaired, broadly in accordance with overall severity [52]. Two patients had mild apraxia of speech, while motor speech was intact in the other three.

The next patient, A11, met WAB criteria for conduction aphasia but this did not adequately characterize her, because unlike the other five patients with conduction aphasia, she was markedly agrammatic in production, yet her sentence comprehension was very good. Her particular profile of deficits was not a good fit for any traditional aphasia subtype [17,18], yet was appropriately characterized by the QAB, with the dissociation between expressive and receptive syntax well captured in her scores for grammatical construction and sentence comprehension.

Four patients (A12, A13, A14, A15) presented with anomic aphasia per clinical impression and per WAB subtype. They spanned a narrow continuum of severity. Word comprehension was uniformly good, while sentence comprehension, word finding, grammatical construction, repetition and reading were generally mildly but variably impaired, broadly in accordance with overall severity. The final patient, A16, had largely recovered from her aphasia, was within normal limits per the WAB, and her QAB overall score of 9.70 did not meet the cutoff for aphasia.

Taken together, these patient profiles suggest that the QAB is capable of quantifying individual patterns of impaired and spared domains within and across aphasia subtypes. However, it must be acknowledged that this evidence regarding the utility of the QAB for characterizing multidimensional profiles is very preliminary in nature. To establish this, it will be necessary to administer the QAB to a much larger sample of patients, and to explicitly test the reliability of the differences between scores on the different summary measures [53].

## Relationships between summary measures

**Fig 15:**
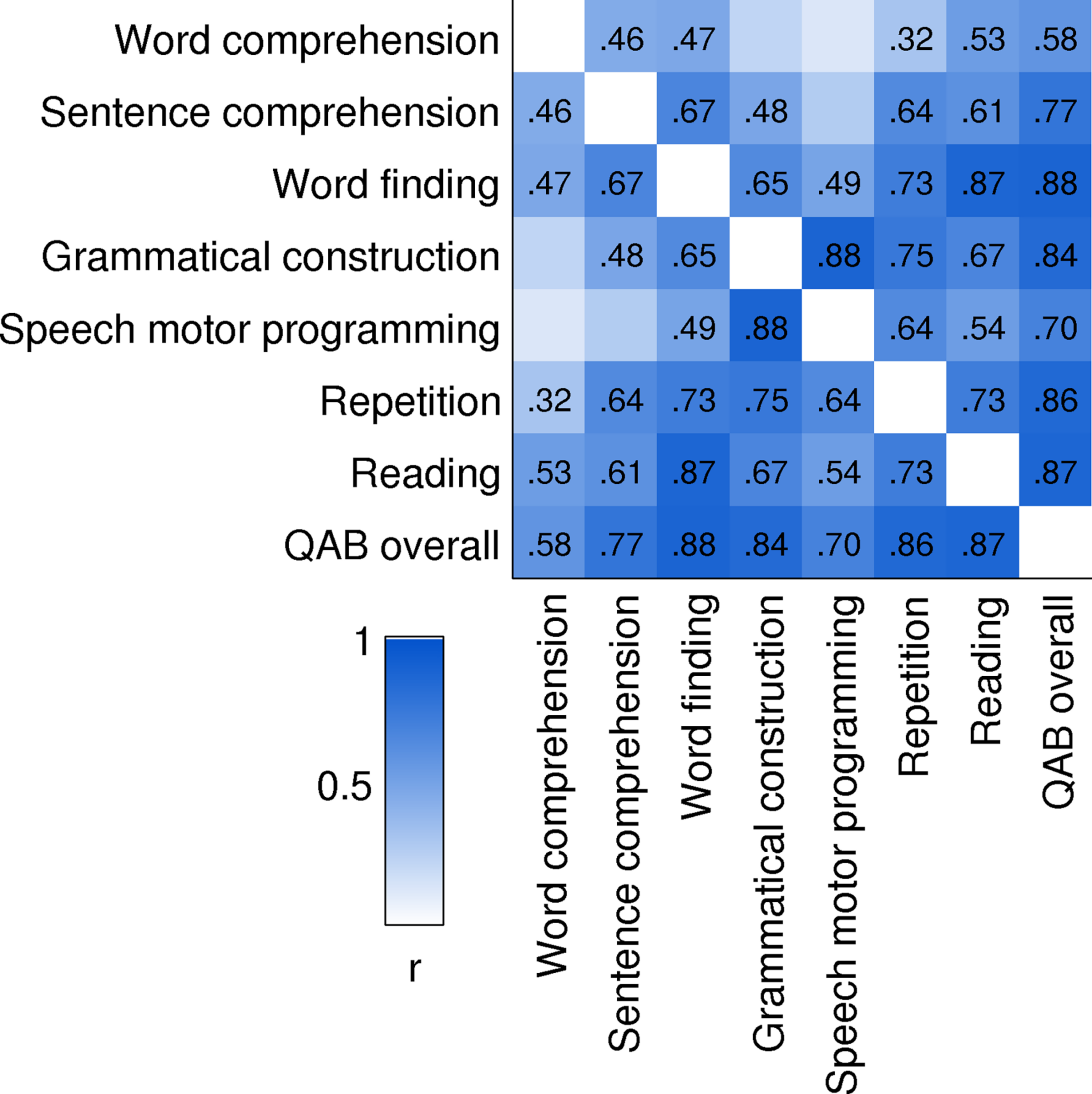
Correlation matrix between QAB measures. Pearson *r* values for significant correlations are shown (*p* < 0.05, uncorrected). Included in this analysis were the 28 patients with acute post-stroke aphasia and the 16 patients with chronic post-stroke aphasia.

A matrix of correlation coefficients was calculated between the eight QAB summary measures, based on the scores of both groups of individuals with aphasia. The chronic group’s scores were averaged across their three sessions, but only the first rater’s scores were used, because only the first rater had also rated the acute patients. The correlation matrix is shown in Fig 15.

Most, but not all, measures correlated with each other. This suggested that an overall severity factor would account for a substantial proportion of the variance, and indeed, principal components analysis showed that the first factor, loading on all summary measures, explained 68% of the variance. But the fact that not all measures were correlated with one another suggests that the summary measures are sensitive to differential impairments in different domains. In particular, it is noteworthy that word comprehension and sentence comprehension were only correlated at *r* = 0.46. Of the three measures that reflect different aspects of fluency, grammatical construction and speech motor programming were highly correlated (*r* = 0.88), but word finding was less strongly correlated with the other two (grammatical construction: *r* = 0.65; speech motor programming: *r* = 0.49).

### Limitations

Several important limitations of the QAB must be noted. First, assessment of written language is minimal: although patients are asked to read words and sentences aloud, reading for comprehension of words or sentences is not tested, nor is writing of words or sentences. This means that deficits specific to written language are not captured. Furthermore, by not testing in the written modality, central deficits may be overestimated in patients with severe auditory input deficits, or severe apraxia of speech; such patients may be able demonstrate retained language abilities via reading or writing. Because of the multiple forms of the QAB, one simple way around this problem would be to test reading and/or writing using items from one of the other forms.

Second, many tradeoffs were made in the interests of reducing administration time. For instance, the six second response time cutoff, and lack of any cueing, may underestimate language abilities in certain patients. Although test-retest reliability was excellent for most measures, it would be desirable for each subtest to include more items, especially the two comprehension subtests.

Third, although the aim of the QAB was to characterize aphasias in terms of summary measures capturing functions known to be neurally and functionally distinct, this was only partially possible in such a brief battery. For example, word comprehension and sentence comprehension are distinguished, yet if there are deficits in the comprehension of single words, this will inevitably impact the sentence comprehension score as well. Another example is the word finding summary measure, to which the biggest contributing subtest is picture naming. While scores on the picture naming task will reflect word finding, they will also be impacted by other functions such as phonemic encoding and even apraxia of speech, if present. This implies that the word finding summary measure will also include contributions of these other functions. Completely disentangling all of the interacting domains of language would require extensive testing, along the lines of the Psycholinguistic Assessments of Language Processing in Aphasia (PALPA) [54], which is unfortunately very time consuming. When the research context dictates a time-efficient evaluation, the best workaround may involve judicious and theoretically motivated analysis of covariance. With an adequate sample size, one could, for instance, covary out apraxia of speech from the word finding summary measure. Related to this general issue, much work remains to be done to characterize the internal structure of the QAB. Although rationales were provided for the calculation of the eight summary measures, a factor analysis of a much larger sample of patients will be necessary to identify underlying factors, and to determine whether the summary measures appropriately reflect the latent factors.

Fourth, the normative data were obtained only from individuals with post-stroke aphasia. Moreover, only three aphasia subtypes (Broca’s, conduction, and anomic) were well represented among the 16 chronic patients who were investigated in more depth. The utility of the QAB in other clinical populations, such as primary progressive aphasia, remains to be investigated.

Finally, the data on inter-rater reliability and test-retest reliability were obtained in chronic, rather than acute stroke patients, even though the time constraints that would motivate the use of a battery such as the QAB are more likely to be applicable at the acute stage. This limitation reflects in part the rapid improvements in language function that often take place in the acute stage [55], which would make it unfeasible to administer the battery on different days in seeking to establish test-retest reliability. Sequential administration of multiple forms in the same session could address this issue, but many acute stroke patients would not tolerate much more than a quarter of an hour of testing. Related to this issue, no evidence has been provided regarding the sensitivity of the QAB to positive changes in language function (due to treated or untreated recovery) or negative changes (due to neurodegeneration).

## Conclusions

This paper has described the construction and validation of the QAB, a new aphasia battery that can typically be administered in about a quarter of an hour, and yields multidimensional profiles of individual patients quantifying their strengths and weaknesses across core language domains. This efficient language assessment was made possible by careful selection of items, a graded scoring system, and a set of summary measures conceptualized in terms of underlying language functions. The reference data show that the QAB is reliable and valid. Inter-rater reliability was excellent for all summary measures. Test-retest reliability was excellent for six summary measures and good for two (word comprehension and sentence comprehension). All of the QAB summary measures corresponded closely to related measures in the WAB (except for reading, which was not assessed in the core WAB), indicating strong concurrent validity. The profiles of 16 chronic post-stroke patients showed that the QAB can reveal individual patterns of spared and impaired language domains.

## Supporting information

S1 Test materials Score-sheets for the three forms of the quick aphasia battery. (PDF)

S2 Test materials Stimulus cards for the three forms of the quick aphasia battery. (PDF)

S1 Macro Macro to calculate summary scores. (XLS)

S1 Dataset Complete scoring of all evaluations. (TXT)
