# Correction: A quick aphasia battery for efficient, reliable, and multidimensional assessment of language function

**DOI:** 10.1371/journal.pone.0199469

**Published:** 2018-06-15

**Authors:** Stephen M. Wilson, Dana K. Eriksson, Sarah M. Schneck, Jillian M. Lucanie

The macro included in the Supporting Information file, S1 Macro, contains several errors that will result in incorrect computations of summary measures. The macro containing the errors was not used in data analysis for the article, and the article’s content is unaffected by these errors. Please see a corrected version of the macro here.

## Supporting information

S1 Macro Macro to calculate summary scores. (XLS)
