# Supplementary material for: A quick aphasia battery for efficient, reliable, and multidimensional assessment of language function
Source: PLoS One. 2018 Feb 9;13(2):e0192773. doi: 10.1371/journal.pone.0192773 (PMC5806902; doi:10.1371/journal.pone.0192773)
Supplement: S1 Test materials — (PDF) [file pone.0192773.s001.pdf]

Quick Aphasia Battery (QAB)

Form 1

Participant ______________________ Date ______________ Time ____________

Location ______________________ Examiner ______________________

- Allow 6 seconds to respond to each item. Correct responses after 3 seconds count as delayed and receive 3 points. Score only material produced in the first 6 seconds, except where a response is initiated before 6 seconds, and then continues.
- Score the first complete response (i.e. not a false start, nor a fragment).
- Verbally presented items may be repeated once, if the patient requests, or if the examiner suspects mishearing or a lapse in attention. If an item is repeated, restart the 6 second count.
- Dysarthric or apraxic errors not affecting the identity of any phonemes should be ignored, except when rating motor speech.

1. Level of consciousness

Can be approached? Score as follows: 4 = yes; 0 = no, based on prior discussion with nurse(s), doctor(s), etc.

(a) *Is patient sufficiently clinically stable to be approached?* [0 **(stop)**] [4]

Can be roused? Score as follows: 4 = already awake or rouses to mild stimulation; 3 = rouses to moderate stimulation; 2 = rouses, but does not stay awake; 1 = shows some response but does not rouse; 0 = no response.

(b) *Can patient be roused?* [0 **(stop)**] [1 **(stop)**] [2] [3] [4]

Oriented? Score as follows: 4 = an intelligible, correct answer (any speech/language errors may be ignored here); 3 = correct answers to all three yes/no questions; 2 = correct on 'yes' question and correct on 1/2 'no' questions; 1 = equivocal signs of understanding and attempting to respond; 0 = response shows no sign of understanding the question, or no response.

(c) Can you tell me where we are right now? ______________________ [0] [1] [2] [3] [4]

If NR/incorrect:	Are we in a library?	[yes] [no] [NR]
	Are we at a playground?	[yes] [no] [NR]
	Are we at [correct place]?	[yes] [no] [NR]

(d) What month is it? ______________________ [0] [1] [2] [3] [4]

If NR/incorrect:	Is it [wrong month]?	[yes] [no] [NR]
	Is it [wrong month]?	[yes] [no] [NR]
	Is it [correct month]?	[yes] [no] [NR]

(e) How old are you? ______________________ [0] [1] [2] [3] [4]

If NR/incorrect:	Are you [wrong age]?	[yes] [no] [NR]
	Are you [correct age]?	[yes] [no] [NR]
	Are you [wrong age]?	[yes] [no] [NR]

Can follow commands? Score as follows: 4 = follows verbal command; 3 = imitates action; 1 = equivocal attempt to follow verbal command or imitate action; 0 = response shows no sign of understanding the command, or no response. Alternative simple commands may be substituted if situational or patient factors dictate, in which case they should be noted.

(f) Close your eyes. ______________________

If not done: *Model closing eyes, indicate to copy.* [0] [1] [3] [4]

(g) Squeeze my hand. ______________________

If not done: *Squeeze patient's hand, indicate to copy.* [0] [1] [3] [4]

Overall impression. Score as follows: 4 = yes; 3 = yes, with reservations (e.g. some lapses); 2 = on and off; 1 = marginally; 0 = not at all.

(h) *Can patient stay awake, maintain attention, and attempt commands?*

[0 **(stop)**] [1 **(stop)**] [2 **(stop)**] [3] [4]

2. Connected speech

Converse with the participant for at least three minutes, around one or more conversation topics, e.g.:

– the best trip you ever took
– your favorite holiday as a child
– your worst childhood memory
– when you retired
– the worst trip you ever took
– a happy childhood memory
– when you had your first child
– what you like about where you live
– when you got married
– your first job
– how you met your husband/wife/partner
– a time you were really scared/embarrassed/angry

____

____

____

____

____

Present stimulus card 1. Ask **'What is happening here?'**

(a) The boy is pushing the girl. ____________________________

(b) The girl is chasing the boy. ____________________________

Rate connected speech using the following scales. Other connected speech produced in the evaluation should also be considered. 4 = normal: not present or within normal range; 3 = mild: detectable but infrequent; 2= moderate: frequent but not pervasive; 1 = marked: pervasive but not ubiquitous; 0 = severe: evident in most or all utterances.

**Aphasic connected speech feature**	**0**	**1**	**2**	**3**	**4**
Reduced length and complexity of utterances	single words	MLU 2 words	MLU 3-4 words	MLU 5-7 words	normal
Reduced speech rate	0-24 wpm	25-49 wpm	50-74 wpm	75-99 wpm	100+ wpm
Agrammatism: omission of words and morphemes, especially closed class; "telegraphic speech"	severe	marked	moderate	mild	normal
Paragrammatism: substitution of closed class items; inappropriate juxtaposition of words and phrases; garbled syntax	severe	marked	moderate	mild	normal
Anomia: word-finding pauses, word-finding failures, abandoned utterances, effective circumlocution	severe	marked	moderate	mild	normal
Empty speech: vague words, overabundance of closed class words, ineffective circumlocution, utterances unclear in meaning and/or tangential	severe	marked	moderate	mild	normal
Semantic paraphasias: substitution of open class items (including perseverations)	severe	marked	moderate	mild	normal
Phonemic paraphasias and neologisms	severe	marked	moderate	mild	normal
Self-correction: false starts, retraced sequences, conduite d'approche	severe	marked	moderate	mild	normal
Overall communication impairment	communication fragmentary; examiner carries burden	communication limited; participant shares burden	can discuss everyday topics; limited beyond that	impairment evident, but can discuss all topics	no impairment evident

If not possible: [ ] No spontaneous speech [ ] Only incomprehensible muttering [ ] Only stereotypies
[ ] Fewer than 10 wpm, typically mainly 'yes', 'no', a few single words or attempts

3. Word comprehension

Present stimulus card 2. Say **'Show me the...'** Score as follows: 4 = correct; 3 = correct, but delayed >3 seconds, self-corrected, or repeated; 1 = related foil; 0 = unrelated foil, or no response within 6 seconds.

(a) lion	Related: giraffe, horse	______________________	[0] [1] [3] [4]
(b) drum	Related: violin, trombone	______________________	[0] [1] [3] [4]
(c) violin	Related: drum, trombone	______________________	[0] [1] [3] [4]
(d) giraffe	Related: lion, horse	______________________	[0] [1] [3] [4]

Present stimulus card 3.

(e) bone	Related: boat	______________________	[0] [1] [3] [4]
(f) kite	Related: coat	______________________	[0] [1] [3] [4]
(g) boot	Related: boat	______________________	[0] [1] [3] [4]
(h) goat	Related: coat	______________________	[0] [1] [3] [4]

4. Sentence comprehension

Say **'Answer yes or no...'** Score as follows: 4 = correct. Nodding and shaking the head, other contextually appropriate alternatives to yes/no, e.g. 'sometimes', 'I don't think so', or idiosyncratic responses that demonstrate understanding are acceptable; 3 = correct, but delayed >3 seconds, self-corrected, or repeated; 2 = idiosyncratic response where it is unclear whether or not patient understands; 1 = incorrect, but response suggests some partial comprehension; 0 = incorrect/no response within 6 seconds.

(a) Are you a [man/woman]? (**Y**)	______________________	[0] [1] [2] [3] [4]
(b) Am I a [man/woman]? (**N**)	______________________	[0] [1] [2] [3] [4]
(c) Do you cut the grass with an axe? (**N**)	______________________	[0] [1] [2] [3] [4]
(d) Are babies watched by babysitters? (**Y**)	______________________	[0] [1] [2] [3] [4]
(e) Do you open your door with a key? (**Y**)	______________________	[0] [1] [2] [3] [4]
(f) If you're about to leave, have you left yet? (**N**)	______________________	[0] [1] [2] [3] [4]
(g) Are witnesses questioned by police? (**Y**)	______________________	[0] [1] [2] [3] [4]
(h) If I tell you I used to smoke, do you think I smoke now? (**N**)	______________________	[0] [1] [2] [3] [4]
(i) Are doctors treated by patients? (**N**)	______________________	[0] [1] [2] [3] [4]
(j) If I was at the park when you arrived, did I get there first? (**Y**)	______________________	[0] [1] [2] [3] [4]
(k) If you're about to go upstairs, are you still downstairs (**Y**)	______________________	[0] [1] [2] [3] [4]
(l) Are cats chased by mice? (**N**)	______________________	[0] [1] [2] [3] [4]

5. Picture naming

Present stimulus card 5. Ask **'What is this? And this? etc.'** Score as follows: 4 = correct. Accept reasonable alternative labels; 3 = correct, but delayed >3 seconds or self-corrected; 2 = at least half of the phonemes are correct, or apraxic error on target; 1 = some relation to target; 0 = unrelated response, or no response within 6 seconds.

(a) dog ______________________ [0] [1] [2] [3] [4]

(b) pencil ______________________ [0] [1] [2] [3] [4]

(c) wheelchair ______________________ [0] [1] [2] [3] [4]

(d) octopus ______________________ [0] [1] [2] [3] [4]

(e) hammock ______________________ [0] [1] [2] [3] [4]

(f) escalator ______________________ [0] [1] [2] [3] [4]

6. Repetition

Say **'Repeat after me.'** Score as follows: 4 = correct. Expanding contraction in (f) is acceptable; 3 = correct, but delayed >3 seconds, self-corrected, or repeated; 2 = (a-d) at least half of the phonemes are correct, or apraxic error on target; (e-f) at least half of the words are correct; 1 = some relation to target, 2+ words for (e) and (f); 0 = unrelated response, or no response within 6 seconds.

(a) house ______________________ [0] [1] [2] [3] [4]

(b) breakfast ______________________ [0] [1] [2] [3] [4]

(c) catastrophe ______________________ [0] [1] [2] [3] [4]

(d) undetectable ______________________ [0] [1] [2] [3] [4]

(e) The sun rises in the East. ______________________ [0] [1] [2] [3] [4]

(f) The ambitious journalist discovered where we'd be going. [0] [1] [2] [3] [4]

____

7. Reading aloud

Present stimulus card 6. Say **'Read these words and sentences out loud.'** Score as follows: 4 = correct. Expanding contraction in (f) is acceptable; 3 = correct, but delayed >3 seconds, self-corrected, or repeated; 2 = (a-d) at least half of the phonemes are correct, or apraxic error on target; (e-f) at least half of the words are correct; 1 = some relation to target, 2+ words for (e) and (f); 0 = unrelated response, or no response within 6 seconds.

(a) tin ______________________ [0] [1] [2] [3] [4]

(b) dough ______________________ [0] [1] [2] [3] [4]

(c) proposition ______________________ [0] [1] [2] [3] [4]

(d) inexperienced ______________________ [0] [1] [2] [3] [4]

(e) The baby cries in the night. ______________________ [0] [1] [2] [3] [4]

(f) The popular novelist realized why I'd been calling. [0] [1] [2] [3] [4]

____

8. Motor speech

Ask patient to perform tasks (a), (c) and (d) as quickly as possible. Ask them to hold vowel in (b) as long as possible, up to 15 seconds. Counting can be done at normal rate. If motor speech deficits are suspected, additional items may be given (e.g. pursing and retracting lips, tʌ tʌ tʌ, kʌ kʌ kʌ, catastrophe catastrophe catastrophe, sustained s).

(a) *tongue from side to side* ______________________

(b) aaaaaah (voice quality) ______________________

(c) pʌ pʌ pʌ pʌ pʌ (rate/rhythm) ______________________

(d) pʌtʌkʌ pʌtʌkʌ pʌtʌkʌ (rate/rhythm) ______________________

(e) Count to 10. ______________________

Rate motor speech using the following scales, based on the whole evaluation, not just this section.

**Motor speech feature**	**0**	**1**	**2**	**3**	**4**
Dysarthria: impairment of muscles used for speech production	severe	marked	moderate	mild	normal
Apraxia of speech: impairment of speech motor planning	severe	marked	moderate	mild	normal

Summary

Word comprehension ________

Sentence comprehension ________

Word finding ________

Grammatical construction ________

Speech motor programming ________

Repetition ________

Reading ________

QAB overall ________

Notes

Quick Aphasia Battery (QAB)

Form 2

Participant ______________________ Date ______________ Time ____________

Location ______________________ Examiner ______________________

- Allow 6 seconds to respond to each item. Correct responses after 3 seconds count as delayed and receive 3 points. Score only material produced in the first 6 seconds, except where a response is initiated before 6 seconds, and then continues.
- Score the first complete response (i.e. not a false start, nor a fragment).
- Verbally presented items may be repeated once, if the patient requests, or if the examiner suspects mishearing or a lapse in attention. If an item is repeated, restart the 6 second count.
- Dysarthric or apraxic errors not affecting the identity of any phonemes should be ignored, except when rating motor speech.

1. Level of consciousness

Can be approached? Score as follows: 4 = yes; 0 = no, based on prior discussion with nurse(s), doctor(s), etc.

(a) *Is patient sufficiently clinically stable to be approached?* [0 **(stop)**] [4]

Can be roused? Score as follows: 4 = already awake or rouses to mild stimulation; 3 = rouses to moderate stimulation; 2 = rouses, but does not stay awake; 1 = shows some response but does not rouse; 0 = no response.

(b) *Can patient be roused?* [0 **(stop)**] [1 **(stop)**] [2] [3] [4]

Oriented? Score as follows: 4 = an intelligible, correct answer (any speech/language errors may be ignored here); 3 = correct answers to all three yes/no questions; 2 = correct on 'yes' question and correct on 1/2 'no' questions; 1 = equivocal signs of understanding and attempting to respond; 0 = response shows no sign of understanding the question, or no response.

(c) Can you tell me where we are right now? ______________________ [0] [1] [2] [3] [4]

If NR/incorrect:	Are we in a library?	[yes] <u>[no]</u> [NR]
	Are we at a playground?	[yes] <u>[no]</u> [NR]
	Are we at [correct place]?	<u>[yes]</u> [no] [NR]

(d) What month is it? ______________________ [0] [1] [2] [3] [4]

If NR/incorrect:	Is it [wrong month]?	[yes] <u>[no]</u> [NR]
	Is it [wrong month]?	[yes] <u>[no]</u> [NR]
	Is it [correct month]?	<u>[yes]</u> [no] [NR]

(e) How old are you? ______________________ [0] [1] [2] [3] [4]

If NR/incorrect:	Are you [wrong age]?	[yes] <u>[no]</u> [NR]
	Are you [correct age]?	<u>[yes]</u> [no] [NR]
	Are you [wrong age]?	[yes] <u>[no]</u> [NR]

Can follow commands? Score as follows: 4 = follows verbal command; 3 = imitates action; 1 = equivocal attempt to follow verbal command or imitate action; 0 = response shows no sign of understanding the command, or no response. Alternative simple commands may be substituted if situational or patient factors dictate, in which case they should be noted.

(f) Close your eyes. ______________________

If not done: *Model closing eyes, indicate to copy.* [0] [1] [3] [4]

(g) Squeeze my hand. ______________________

If not done: *Squeeze patient's hand, indicate to copy.* [0] [1] [3] [4]

Overall impression. Score as follows: 4 = yes; 3 = yes, with reservations (e.g. some lapses); 2 = on and off; 1 = marginally; 0 = not at all.

(h) *Can patient stay awake, maintain attention, and attempt commands?*

[0 **(stop)**] [1 **(stop)**] [2 **(stop)**] [3] [4]

2. Connected speech

Converse with the participant for at least three minutes, around one or more conversation topics, e.g.:

– the best trip you ever took
– your favorite holiday as a child
– your worst childhood memory
– when you retired
– the worst trip you ever took
– a happy childhood memory
– when you had your first child
– what you like about where you live
– when you got married
– your first job
– how you met your husband/wife/partner
– a time you were really scared/embarrassed/angry

____

____

____

____

____

Present stimulus card 1. Ask **'What is happening here?'**

(a) The boy is washing the girl. ______________________________

(b) The girl is kicking the boy. ______________________________

Rate connected speech using the following scales. Other connected speech produced in the evaluation should also be considered. 4 = normal: not present or within normal range; 3 = mild: detectable but infrequent; 2= moderate: frequent but not pervasive; 1 = marked: pervasive but not ubiquitous; 0 = severe: evident in most or all utterances.

**Aphasic connected speech feature**	**0**	**1**	**2**	**3**	**4**
Reduced length and complexity of utterances	single words	MLU 2 words	MLU 3-4 words	MLU 5-7 words	normal
Reduced speech rate	0-24 wpm	25-49 wpm	50-74 wpm	75-99 wpm	100+ wpm
Agrammatism: omission of words and morphemes, especially closed class; "telegraphic speech"	severe	marked	moderate	mild	normal
Paragrammatism: substitution of closed class items; inappropriate juxtaposition of words and phrases; garbled syntax	severe	marked	moderate	mild	normal
Anomia: word-finding pauses, word-finding failures, abandoned utterances, effective circumlocution	severe	marked	moderate	mild	normal
Empty speech: vague words, overabundance of closed class words, ineffective circumlocution, utterances unclear in meaning and/or tangential	severe	marked	moderate	mild	normal
Semantic paraphasias: substitution of open class items (including perseverations)	severe	marked	moderate	mild	normal
Phonemic paraphasias and neologisms	severe	marked	moderate	mild	normal
Self-correction: false starts, retraced sequences, conduite d'approche	severe	marked	moderate	mild	normal
Overall communication impairment	communication fragmentary; examiner carries burden	communication limited; participant shares burden	can discuss everyday topics; limited beyond that	impairment evident, but can discuss all topics	no impairment evident

If not possible: [ ] No spontaneous speech [ ] Only incomprehensible muttering [ ] Only stereotypies
[ ] Fewer than 10 wpm, typically mainly 'yes', 'no', a few single words or attempts

3. Word comprehension

Present stimulus card 2. Say **'Show me the...'** Score as follows: 4 = correct; 3 = correct, but delayed >3 seconds, self-corrected, or repeated; 1 = related foil; 0 = unrelated foil, or no response within 6 seconds.

(a)	guitar	Related: saxophone, harp	____________	[0] [1] [3] [4]
(b)	tiger	Related: zebra, donkey	____________	[0] [1] [3] [4]
(c)	zebra	Related: tiger, donkey	____________	[0] [1] [3] [4]
(d)	saxophone	Related: guitar, harp	____________	[0] [1] [3] [4]

Present stimulus card 3.

(e)	pear	Related: chair, hair	____________	[0] [1] [3] [4]
(f)	crown	Related: clown	____________	[0] [1] [3] [4]
(g)	cloud	Related: clown	____________	[0] [1] [3] [4]
(h)	chair	Related: pear, hair	____________	[0] [1] [3] [4]

4. Sentence comprehension

Say **'Answer yes or no...'** Score as follows: 4 = correct. Nodding and shaking the head, other contextually appropriate alternatives to yes/no, e.g. 'sometimes', 'I don't think so', or idiosyncratic responses that demonstrate understanding are acceptable; 3 = correct, but delayed >3 seconds, self-corrected, or repeated; 2 = idiosyncratic response where it is unclear whether or not patient understands; 1 = incorrect, but response suggests some partial comprehension; 0 = incorrect/no response within 6 seconds.

(a)	Are you [sitting/lying down/etc.]? (**Y**)	____________	[0] [1] [2] [3] [4]
(b)	Am I [sitting/standing/etc.]? (**N**)	____________	[0] [1] [2] [3] [4]
(c)	Do you eat ice cream with a spoon? (**Y**)	____________	[0] [1] [2] [3] [4]
(d)	Are spiders bitten by people? (**N**)	____________	[0] [1] [2] [3] [4]
(e)	Do you wear gloves on your feet? (**N**)	____________	[0] [1] [2] [3] [4]
(f)	If you're about to go outside, are you still inside? (**Y**)	____________	[0] [1] [2] [3] [4]
(g)	Are worms eaten by birds? (**Y**)	____________	[0] [1] [2] [3] [4]
(h)	If I tell you I used to exercise, do you think I exercise now? (**N**)	____________	[0] [1] [2] [3] [4]
(i)	Are babies delivered by doctors? (**Y**)	____________	[0] [1] [2] [3] [4]
(j)	If you're about to start, have you started yet? (**N**)	____________	[0] [1] [2] [3] [4]
(k)	Are parents raised by children? (**N**)	____________	[0] [1] [2] [3] [4]
(l)	If you were at the party when I arrived, did you get there first? (**Y**)	____________	[0] [1] [2] [3] [4]

5. Picture naming

Present stimulus card 5. Ask **'What is this? And this? etc.'** Score as follows: 4 = correct. Accept reasonable alternative labels; 3 = correct, but delayed >3 seconds or self-corrected; 2 = at least half of the phonemes are correct, or apraxic error on target; 1 = some relation to target; 0 = unrelated response, or no response within 6 seconds.

(a) book ________________________ [0] [1] [2] [3] [4]

(b) comb ________________________ [0] [1] [2] [3] [4]

(c) mask ________________________ [0] [1] [2] [3] [4]

(d) volcano ________________________ [0] [1] [2] [3] [4]

(e) seahorse ________________________ [0] [1] [2] [3] [4]

(f) pyramid ________________________ [0] [1] [2] [3] [4]

6. Repetition

Say **'Repeat after me.'** Score as follows: 4 = correct. Expanding contraction in (f) is acceptable; 3 = correct, but delayed >3 seconds, self-corrected, or repeated; 2 = (a-d) at least half of the phonemes are correct, or apraxic error on target; (e-f) at least half of the words are correct; 1 = some relation to target, 2+ words for (e) and (f); 0 = unrelated response, or no response within 6 seconds.

(a) man ________________________ [0] [1] [2] [3] [4]

(b) blanket ________________________ [0] [1] [2] [3] [4]

(c) prosperity ________________________ [0] [1] [2] [3] [4]

(d) insignificant ________________________ [0] [1] [2] [3] [4]

(e) The dog barks at the door. ________________________ [0] [1] [2] [3] [4]

(f) The creative architect understood who we'd be seeing. [0] [1] [2] [3] [4]

____

7. Reading aloud

Present stimulus card 6. Say **'Read these words and sentences out loud.'** Score as follows: 4 = correct. Expanding contraction in (f) is acceptable; 3 = correct, but delayed >3 seconds, self-corrected, or repeated; 2 = (a-d) at least half of the phonemes are correct, or apraxic error on target; (e-f) at least half of the words are correct; 1 = some relation to target, 2+ words for (e) and (f); 0 = unrelated response, or no response within 6 seconds.

(a) pig ________________________ [0] [1] [2] [3] [4]

(b) choir ________________________ [0] [1] [2] [3] [4]

(c) graduation ________________________ [0] [1] [2] [3] [4]

(d) involuntary ________________________ [0] [1] [2] [3] [4]

(e) The sun sets in the West. ________________________ [0] [1] [2] [3] [4]

(f) The capable detective discovered why I'd been waiting. [0] [1] [2] [3] [4]

____

8. Motor speech

Ask patient to perform tasks (a), (c) and (d) as quickly as possible. Ask them to hold vowel in (b) as long as possible, up to 15 seconds. Counting can be done at normal rate. If motor speech deficits are suspected, additional items may be given (e.g. pursing and retracting lips, tʌ tʌ tʌ, kʌ kʌ kʌ, catastrophe catastrophe catastrophe, sustained s).

(a) *tongue from side to side* ________________________

(b) aaaaaah (voice quality) ________________________

(c) pʌ pʌ pʌ pʌ pʌ (rate/rhythm) ________________________

(d) pʌtʌkʌ pʌtʌkʌ pʌtʌkʌ (rate/rhythm) ________________________

(e) Count to 10. ________________________

Rate motor speech using the following scales, based on the whole evaluation, not just this section.

**Motor speech feature**	**0**	**1**	**2**	**3**	**4**
Dysarthria: impairment of muscles used for speech production	severe	marked	moderate	mild	normal
Apraxia of speech: impairment of speech motor planning	severe	marked	moderate	mild	normal

Summary

Word comprehension ________

Sentence comprehension ________

Word finding ________

Grammatical construction ________

Speech motor programming ________

Repetition ________

Reading ________

QAB overall ________

Notes

Quick Aphasia Battery (QAB)

Form 3

Participant ______________________ Date ______________ Time ____________

Location ______________________ Examiner ______________________

- Allow 6 seconds to respond to each item. Correct responses after 3 seconds count as delayed and receive 3 points. Score only material produced in the first 6 seconds, except where a response is initiated before 6 seconds, and then continues.
- Score the first complete response (i.e. not a false start, nor a fragment).
- Verbally presented items may be repeated once, if the patient requests, or if the examiner suspects mishearing or a lapse in attention. If an item is repeated, restart the 6 second count.
- Dysarthric or apraxic errors not affecting the identity of any phonemes should be ignored, except when rating motor speech.

1. Level of consciousness

Can be approached? Score as follows: 4 = yes; 0 = no, based on prior discussion with nurse(s), doctor(s), etc.

(a) *Is patient sufficiently clinically stable to be approached?* [0 **(stop)**] [4]

Can be roused? Score as follows: 4 = already awake or rouses to mild stimulation; 3 = rouses to moderate stimulation; 2 = rouses, but does not stay awake; 1 = shows some response but does not rouse; 0 = no response.

(b) *Can patient be roused?* [0 **(stop)**] [1 **(stop)**] [2] [3] [4]

Oriented? Score as follows: 4 = an intelligible, correct answer (any speech/language errors may be ignored here); 3 = correct answers to all three yes/no questions; 2 = correct on 'yes' question and correct on 1/2 'no' questions; 1 = equivocal signs of understanding and attempting to respond; 0 = response shows no sign of understanding the question, or no response.

(c) Can you tell me where we are right now? ______________ [0] [1] [2] [3] [4]

If NR/incorrect:	Are we in a library?	[yes] [no] [NR]
	Are we at a playground?	[yes] [no] [NR]
	Are we at [correct place]?	[yes] [no] [NR]

(d) What month is it? ______________ [0] [1] [2] [3] [4]

If NR/incorrect:	Is it [wrong month]?	[yes] [no] [NR]
	Is it [wrong month]?	[yes] [no] [NR]
	Is it [correct month]?	[yes] [no] [NR]

(e) How old are you? ______________ [0] [1] [2] [3] [4]

If NR/incorrect:	Are you [wrong age]?	[yes] [no] [NR]
	Are you [correct age]?	[yes] [no] [NR]
	Are you [wrong age]?	[yes] [no] [NR]

Can follow commands? Score as follows: 4 = follows verbal command; 3 = imitates action; 1 = equivocal attempt to follow verbal command or imitate action; 0 = response shows no sign of understanding the command, or no response. Alternative simple commands may be substituted if situational or patient factors dictate, in which case they should be noted.

(f) Close your eyes. ______________

If not done: *Model closing eyes, indicate to copy.* [0] [1] [3] [4]

(g) Squeeze my hand. ______________

If not done: *Squeeze patient's hand, indicate to copy.* [0] [1] [3] [4]

Overall impression. Score as follows: 4 = yes; 3 = yes, with reservations (e.g. some lapses); 2 = on and off; 1 = marginally; 0 = not at all.

(h) *Can patient stay awake, maintain attention, and attempt commands?*

[0 **(stop)**] [1 **(stop)**] [2 **(stop)**] [3] [4]

2. Connected speech

Converse with the participant for at least three minutes, around one or more conversation topics, e.g.:

– the best trip you ever took
– your favorite holiday as a child
– your worst childhood memory
– when you retired
– the worst trip you ever took
– a happy childhood memory
– when you had your first child
– what you like about where you live
– when you got married
– your first job
– how you met your husband/wife/partner
– a time you were really scared/embarrassed/angry

Present stimulus card 1. Ask **'What is happening here?'**

(a) The girl is pulling the boy. ____________________

(b) The boy is kissing the girl. ____________________

Rate connected speech using the following scales. Other connected speech produced in the evaluation should also be considered. 4 = normal: not present or within normal range; 3 = mild: detectable but infrequent; 2= moderate: frequent but not pervasive; 1 = marked: pervasive but not ubiquitous; 0 = severe: evident in most or all utterances.

**Aphasic connected speech feature**	**0**	**1**	**2**	**3**	**4**
Reduced length and complexity of utterances	single words	MLU 2 words	MLU 3-4 words	MLU 5-7 words	normal
Reduced speech rate	0-24 wpm	25-49 wpm	50-74 wpm	75-99 wpm	100+ wpm
Agrammatism: omission of words and morphemes, especially closed class; "telegraphic speech"	severe	marked	moderate	mild	normal
Paragrammatism: substitution of closed class items; inappropriate juxtaposition of words and phrases; garbled syntax	severe	marked	moderate	mild	normal
Anomia: word-finding pauses, word-finding failures, abandoned utterances, effective circumlocution	severe	marked	moderate	mild	normal
Empty speech: vague words, overabundance of closed class words, ineffective circumlocution, utterances unclear in meaning and/or tangential	severe	marked	moderate	mild	normal
Semantic paraphasias: substitution of open class items (including perseverations)	severe	marked	moderate	mild	normal
Phonemic paraphasias and neologisms	severe	marked	moderate	mild	normal
Self-correction: false starts, retraced sequences, conduite d'approche	severe	marked	moderate	mild	normal
Overall communication impairment	communication fragmentary; examiner carries burden	communication limited; participant shares burden	can discuss everyday topics; limited beyond that	impairment evident, but can discuss all topics	no impairment evident

If not possible: [ ] No spontaneous speech [ ] Only incomprehensible muttering [ ] Only stereotypies
[ ] Fewer than 10 wpm, typically mainly 'yes', 'no', a few single words or attempts

3. Word comprehension

Present stimulus card 2. Say **'Show me the...'** Score as follows: 4 = correct; 3 = correct, but delayed >3 seconds, self-corrected, or repeated; 1 = related foil; 0 = unrelated foil, or no response within 6 seconds.

(a) elephant — Related: camel, bear — ______________ [0] [1] [3] [4]

(b) piano — Related: trumpet, cello — ______________ [0] [1] [3] [4]

(c) camel — Related: elephant, bear — ______________ [0] [1] [3] [4]

(d) trumpet — Related: piano, cello — ______________ [0] [1] [3] [4]

Present stimulus card 3.

(e) cat — Related: can — ______________ [0] [1] [3] [4]

(f) box — Related: socks, fox — ______________ [0] [1] [3] [4]

(g) cane — Related: can — ______________ [0] [1] [3] [4]

(h) socks — Related: box, fox — ______________ [0] [1] [3] [4]

4. Sentence comprehension

Say **'Answer yes or no...'** Score as follows: 4 = correct. Nodding and shaking the head, other contextually appropriate alternatives to yes/no, e.g. 'sometimes', 'I don't think so', or idiosyncratic responses that demonstrate understanding are acceptable; 3 = correct, but delayed >3 seconds, self-corrected, or repeated; 2 = idiosyncratic response where it is unclear whether or not patient understands; 1 = incorrect, but response suggests some partial comprehension; 0 = incorrect/no response within 6 seconds.

(a) Am I wearing a [color] [shirt/dress]? (**Y**) ______________ [0] [1] [2] [3] [4]

(b) Are you wearing a [color] [shirt/dress]? (**N**) ______________ [0] [1] [2] [3] [4]

(c) Do you brush your teeth with a comb? (**N**) ______________ [0] [1] [2] [3] [4]

(d) Are babies named by parents? (**Y**) ______________ [0] [1] [2] [3] [4]

(e) Do you take pictures with a camera? (**Y**) ______________ [0] [1] [2] [3] [4]

(f) If you're about to finish, have you finished yet? (**N**) ______________ [0] [1] [2] [3] [4]

(g) Are people taxed by governments? (**Y**) ______________ [0] [1] [2] [3] [4]

(h) If you're about to go inside, are you still outside? (**Y**) ______________ [0] [1] [2] [3] [4]

(i) Are wolves attacked by deer? (**N**) ______________ [0] [1] [2] [3] [4]

(j) If he was at the show when you arrived, did he get there first? (**Y**) ______________ [0] [1] [2] [3] [4]

(k) Are thieves robbed by victims? (**N**) ______________ [0] [1] [2] [3] [4]

(l) If I tell you I used to drink coffee, do you think I drink coffee now? (**N**) ______________ [0] [1] [2] [3] [4]

5. Picture naming

Present stimulus card 5. Ask **'What is this? And this? etc.'** Score as follows: 4 = correct. Accept reasonable alternative labels; 3 = correct, but delayed >3 seconds or self-corrected; 2 = at least half of the phonemes are correct, or apraxic error on target; 1 = some relation to target; 0 = unrelated response, or no response within 6 seconds.

(a) bed ______________________ [0] [1] [2] [3] [4]

(b) flower ______________________ [0] [1] [2] [3] [4]

(c) mushroom ______________________ [0] [1] [2] [3] [4]

(d) harmonica ______________________ [0] [1] [2] [3] [4]

(e) pelican ______________________ [0] [1] [2] [3] [4]

(f) stethoscope ______________________ [0] [1] [2] [3] [4]

6. Repetition

Say **'Repeat after me.'** Score as follows: 4 = correct. Expanding contraction in (f) is acceptable; 3 = correct, but delayed >3 seconds, self-corrected, or repeated; 2 = (a-d) at least half of the phonemes are correct, or apraxic error on target; (e-f) at least half of the words are correct; 1 = some relation to target, 2+ words for (e) and (f); 0 = unrelated response, or no response within 6 seconds.

(a) head ______________________ [0] [1] [2] [3] [4]

(b) brother ______________________ [0] [1] [2] [3] [4]

(c) proximity ______________________ [0] [1] [2] [3] [4]

(d) inconceivable ______________________ [0] [1] [2] [3] [4]

(e) The baby drinks from a bottle. ______________________ [0] [1] [2] [3] [4]

(f) The confident vocalist realized where we'd be staying. [0] [1] [2] [3] [4]

____

7. Reading aloud

Present stimulus card 6. Say **'Read these words and sentences out loud.'** Score as follows: 4 = correct. Expanding contraction in (f) is acceptable; 3 = correct, but delayed >3 seconds, self-corrected, or repeated; 2 = (a-d) at least half of the phonemes are correct, or apraxic error on target; (e-f) at least half of the words are correct; 1 = some relation to target, 2+ words for (e) and (f); 0 = unrelated response, or no response within 6 seconds.

(a) pot ______________________ [0] [1] [2] [3] [4]

(b) cough ______________________ [0] [1] [2] [3] [4]

(c) prohibition ______________________ [0] [1] [2] [3] [4]

(d) unforgettable ______________________ [0] [1] [2] [3] [4]

(e) The dog sleeps on the floor. ______________________ [0] [1] [2] [3] [4]

(f) The ethical accountant understood why I'd been hiding. [0] [1] [2] [3] [4]

____

8. Motor speech

Ask patient to perform tasks (a), (c) and (d) as quickly as possible. Ask them to hold vowel in (b) as long as possible, up to 15 seconds. Counting can be done at normal rate. If motor speech deficits are suspected, additional items may be given (e.g. pursing and retracting lips, tʌ tʌ tʌ, kʌ kʌ kʌ, catastrophe catastrophe catastrophe, sustained s).

(a) *tongue from side to side* ____________________

(b) aaaaaah (voice quality) ____________________

(c) pʌ pʌ pʌ pʌ pʌ (rate/rhythm) ____________________

(d) pʌtʌkʌ pʌtʌkʌ pʌtʌkʌ (rate/rhythm) ____________________

(e) Count to 10. ____________________

Rate motor speech using the following scales, based on the whole evaluation, not just this section.

**Motor speech feature**	**0**	**1**	**2**	**3**	**4**
Dysarthria: impairment of muscles used for speech production	severe	marked	moderate	mild	normal
Apraxia of speech: impairment of speech motor planning	severe	marked	moderate	mild	normal

Summary

Word comprehension __________

Sentence comprehension __________

Word finding __________

Grammatical construction __________

Speech motor programming __________

Repetition __________

Reading __________

QAB overall __________

Notes
