# Supplementary material for: A quick aphasia battery for efficient, reliable, and multidimensional assessment of language function
Source: PLoS One. 2018 Feb 9;13(2):e0192773. doi: 10.1371/journal.pone.0192773 (PMC5806902; doi:10.1371/journal.pone.0192773)
Supplement: S2 Test materials — (PDF) [file pone.0192773.s002.pdf]

Quick Aphasia Battery

Form 1

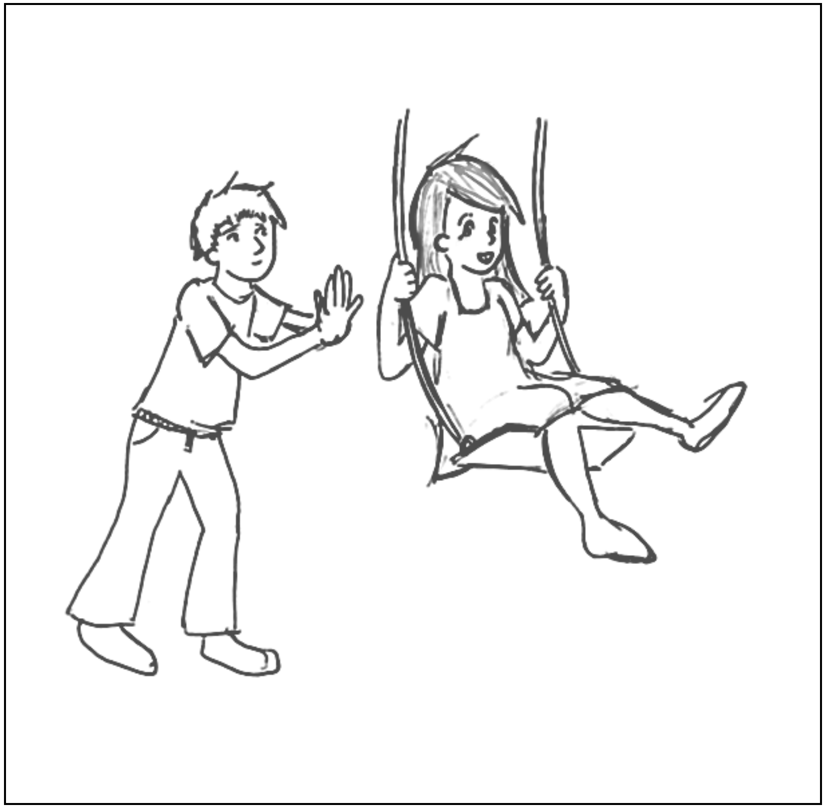

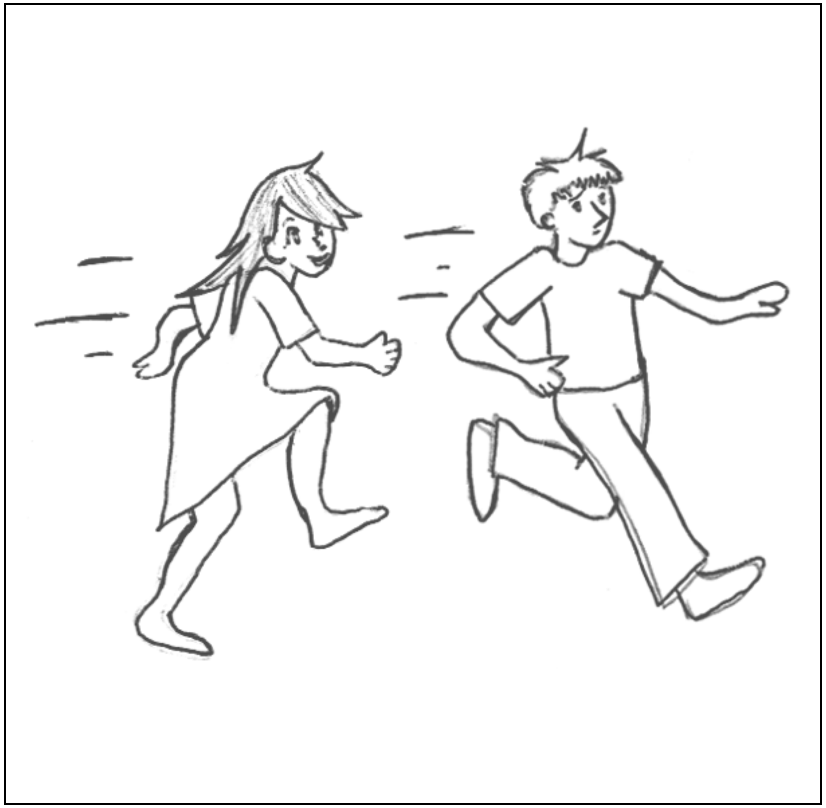

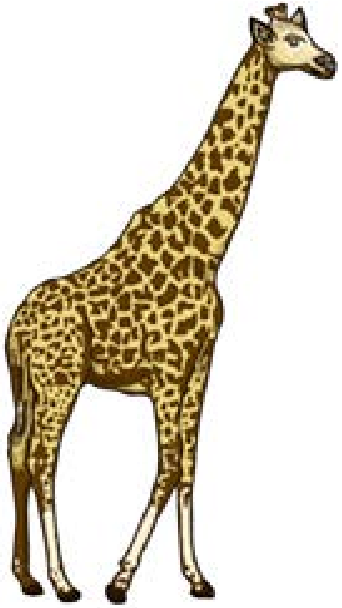
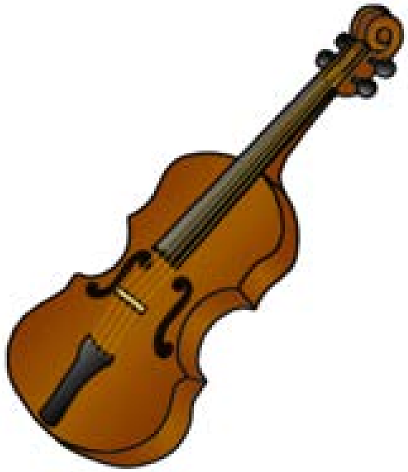
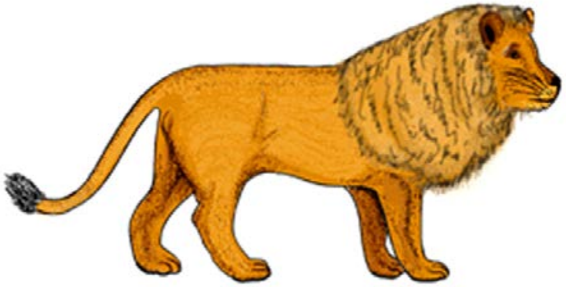
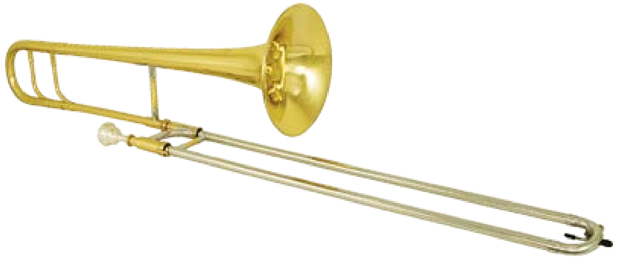
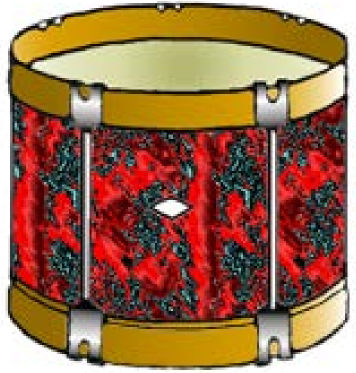
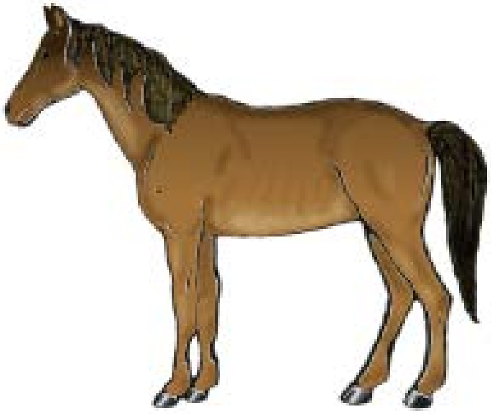

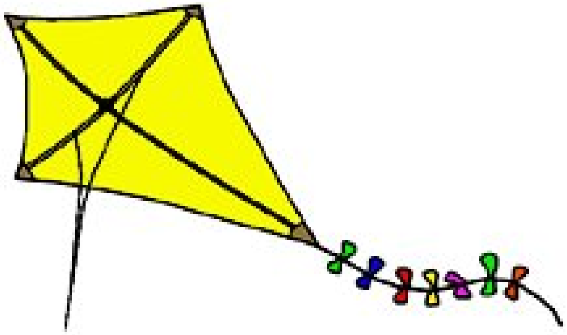
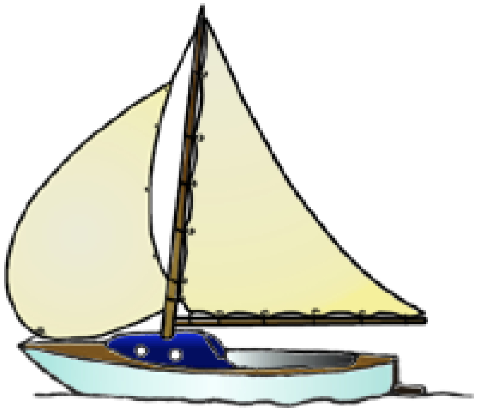
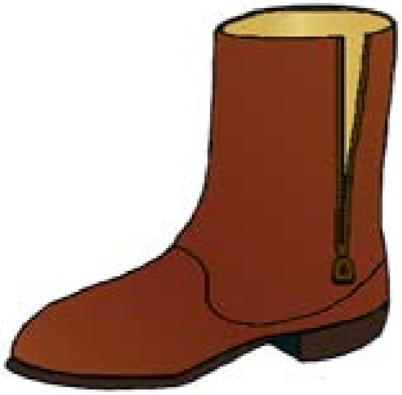
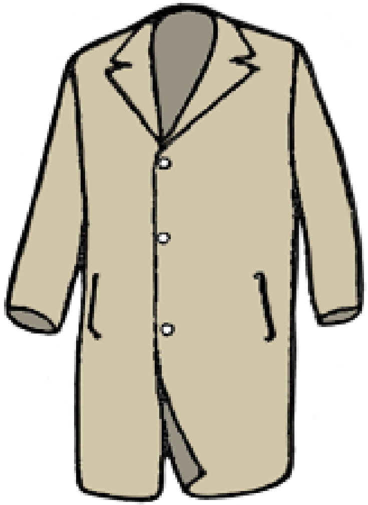
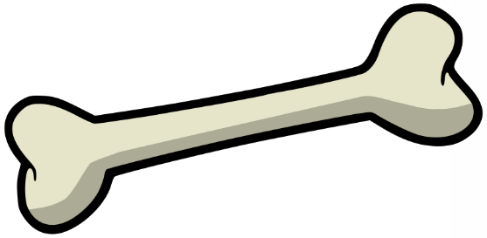
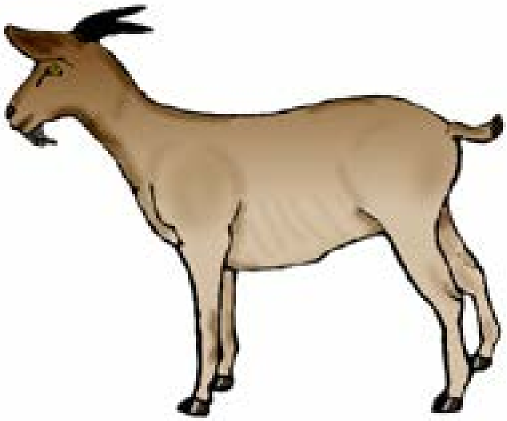

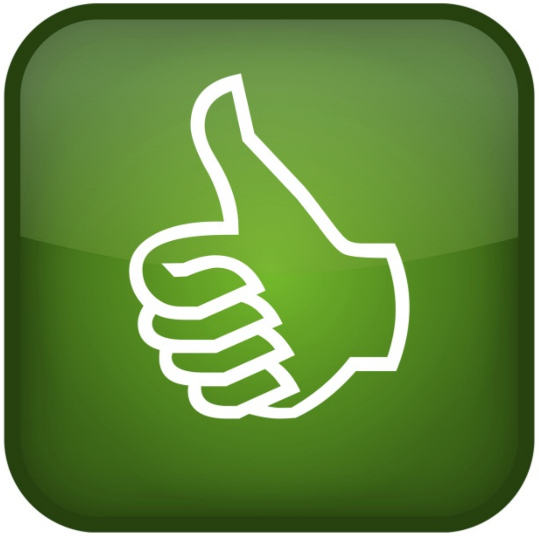

Yes

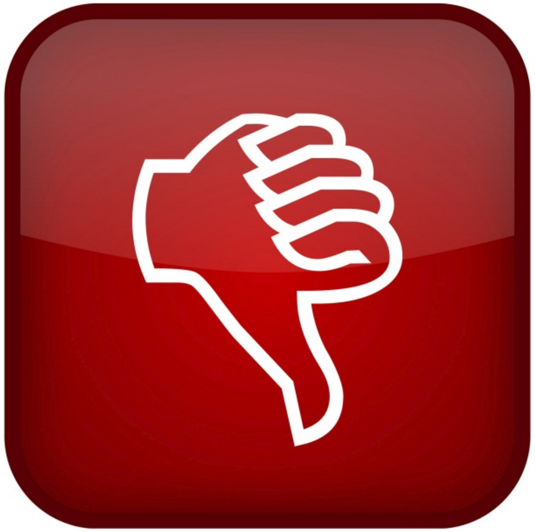

No

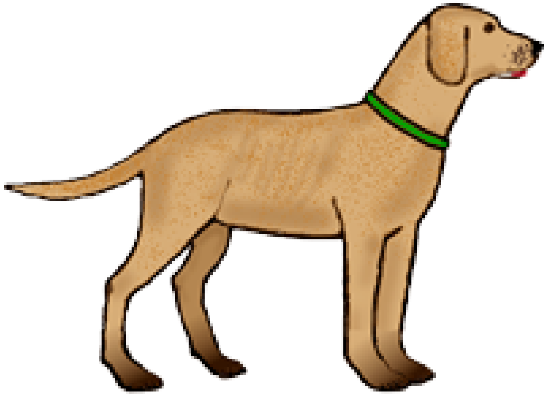
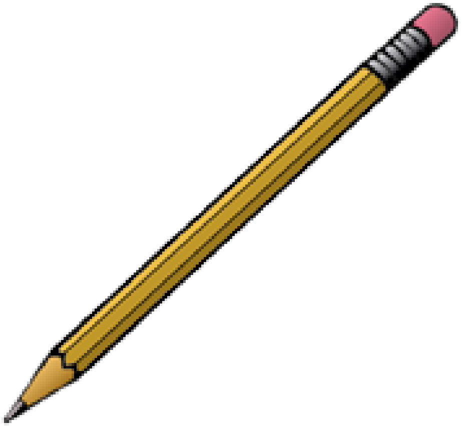
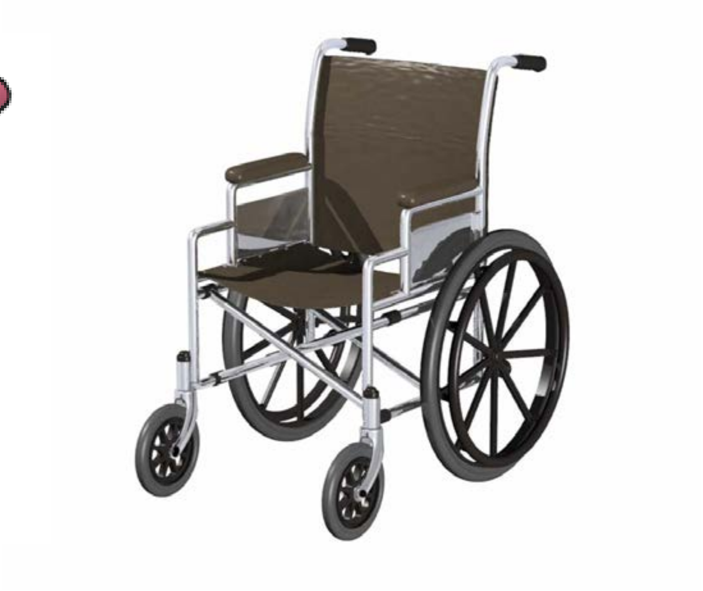
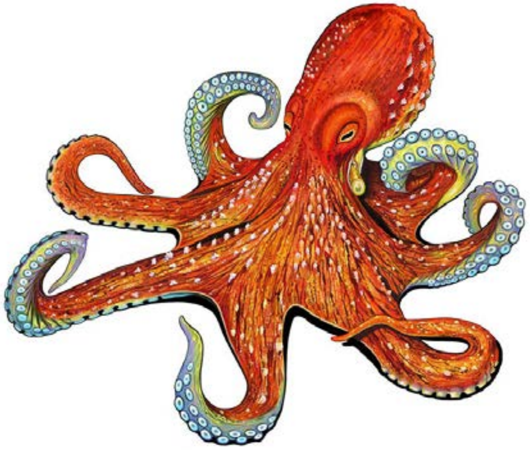
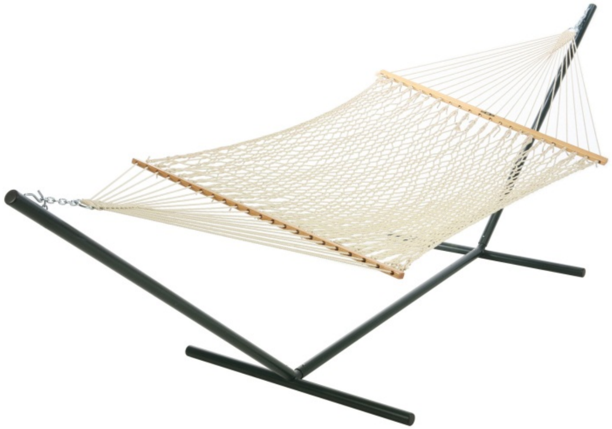
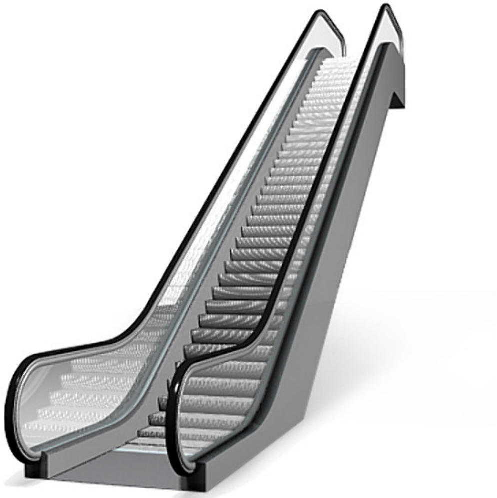

tin

dough

proposition

inexperienced

The baby cries in the night.

The popular novelist realized
why I’d been calling.

Quick Aphasia Battery

Form 2

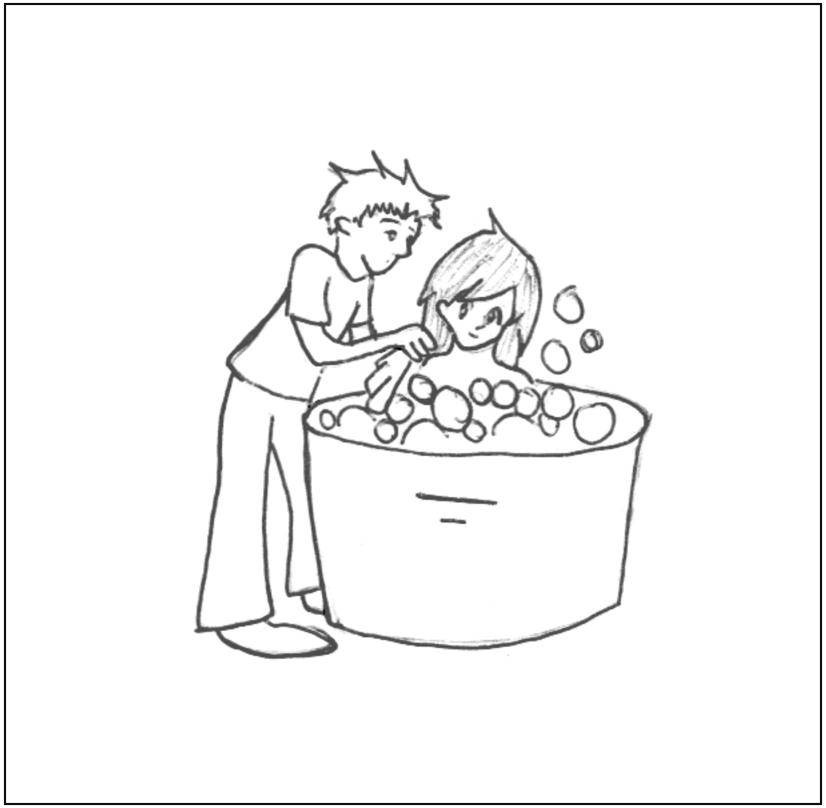

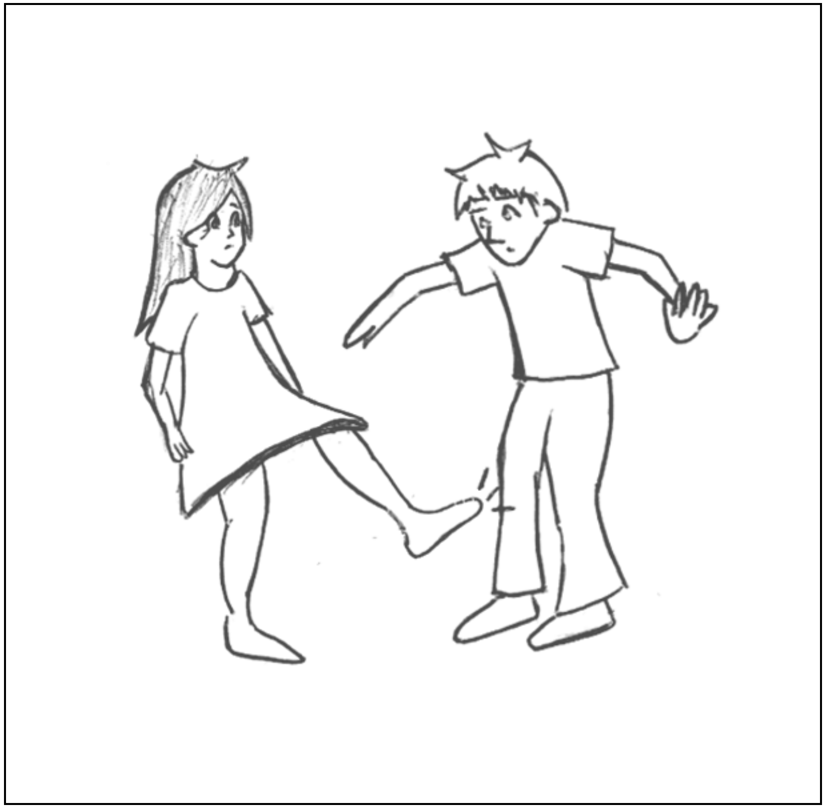

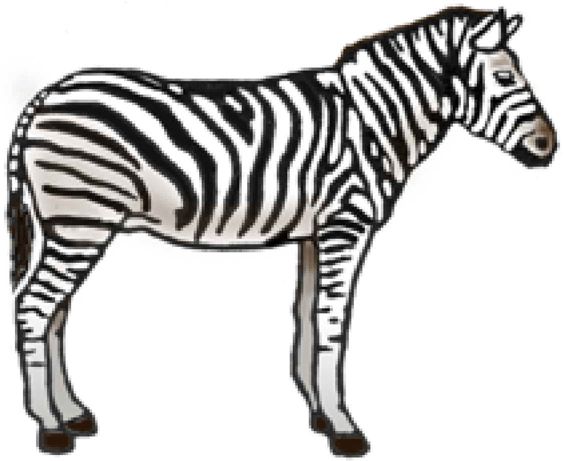
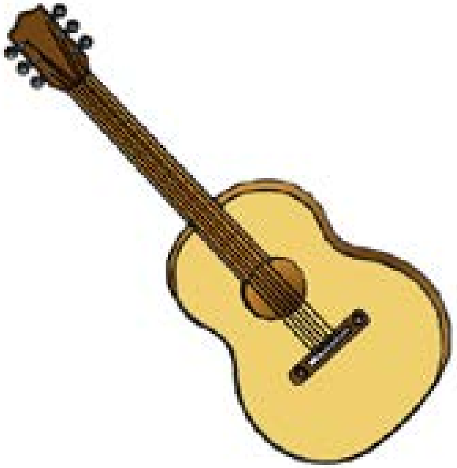
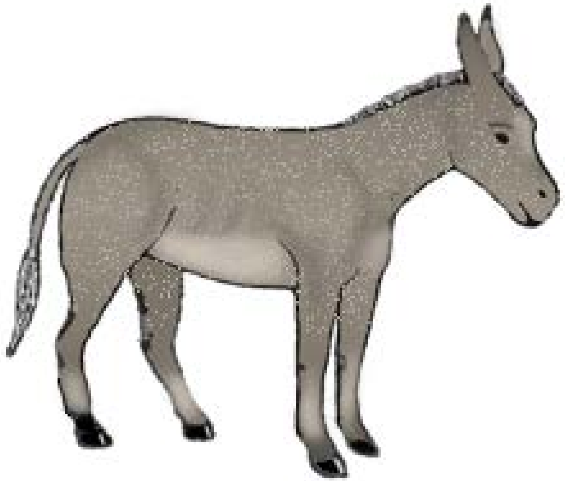
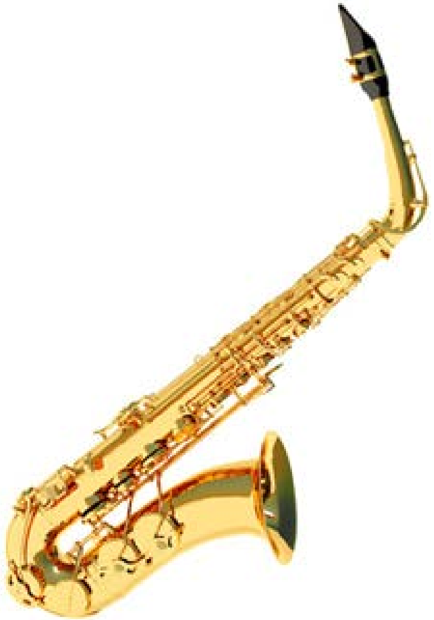
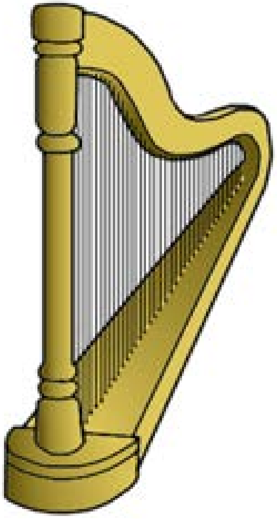
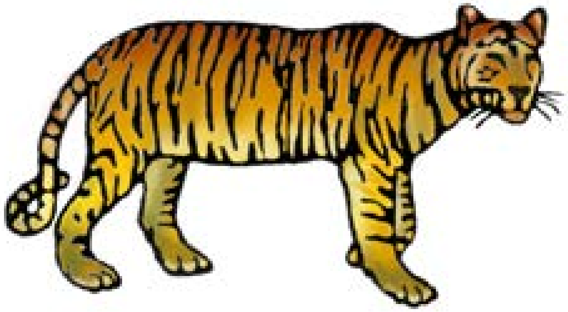

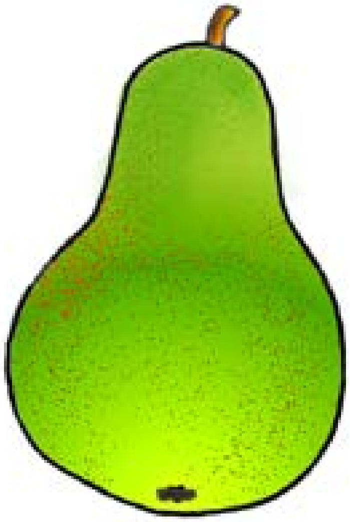
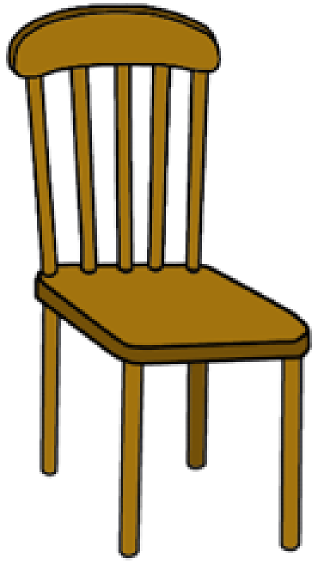
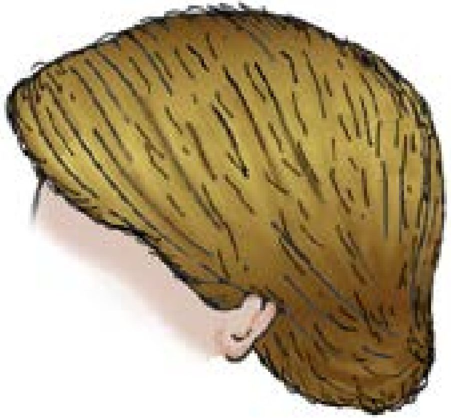
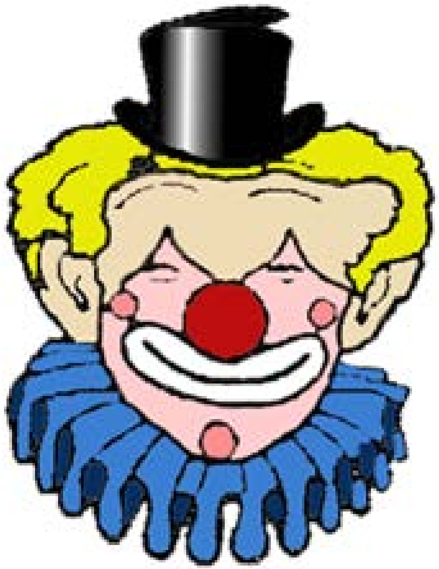
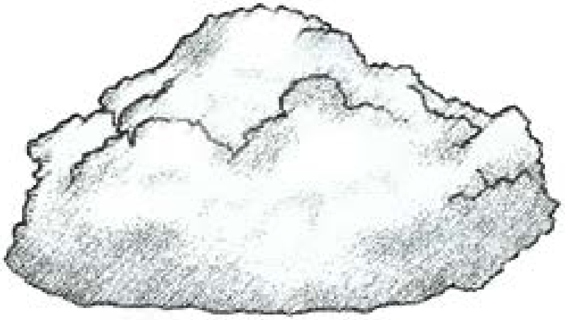
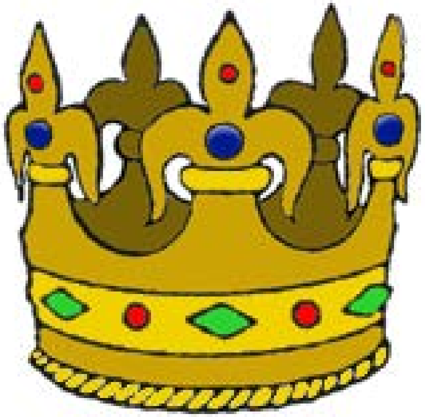

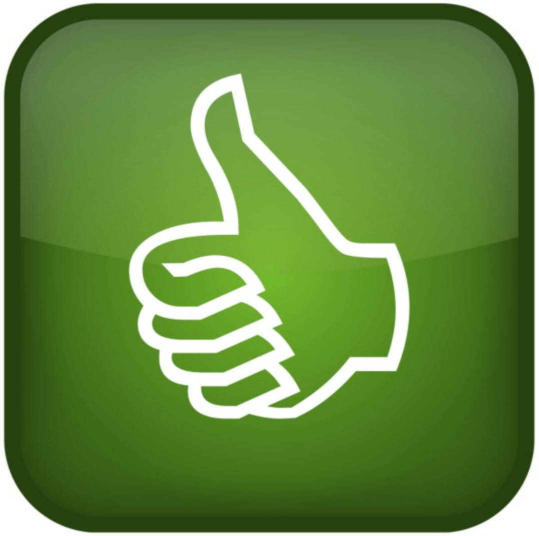

Yes

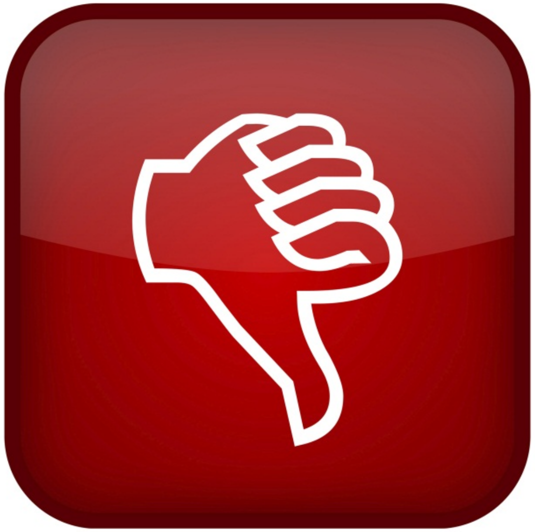

No

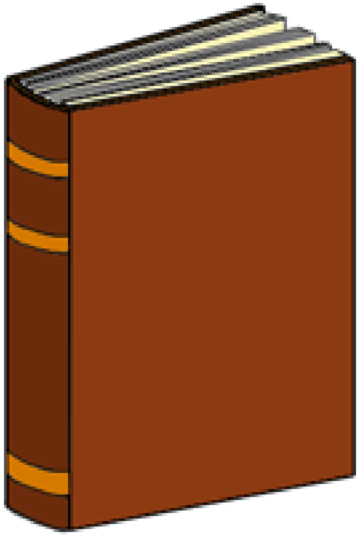
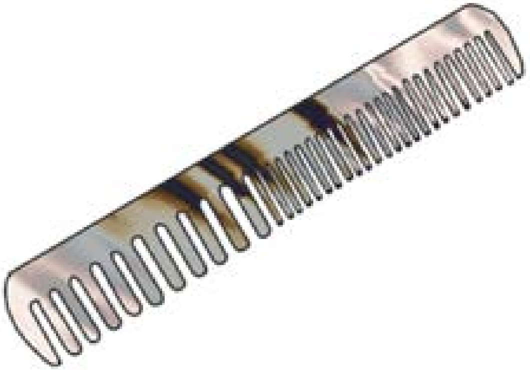
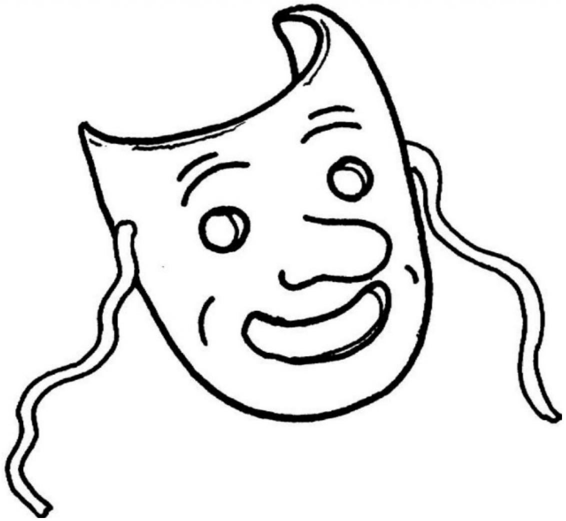
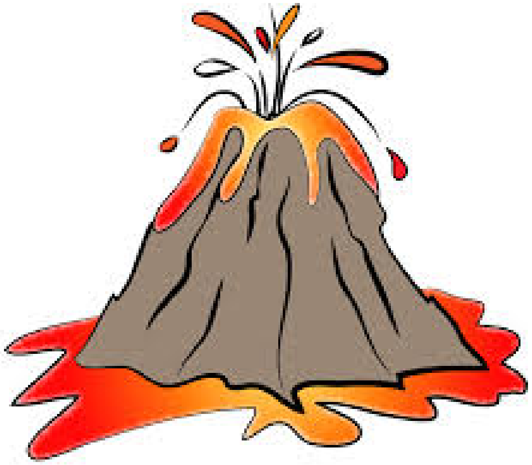
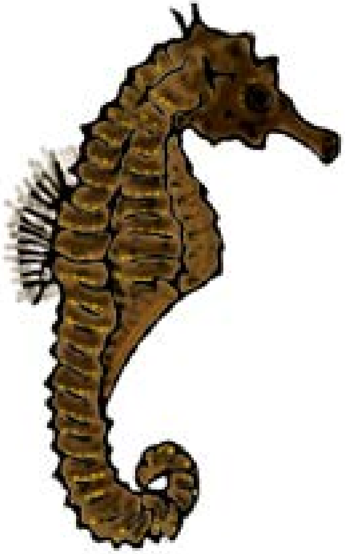
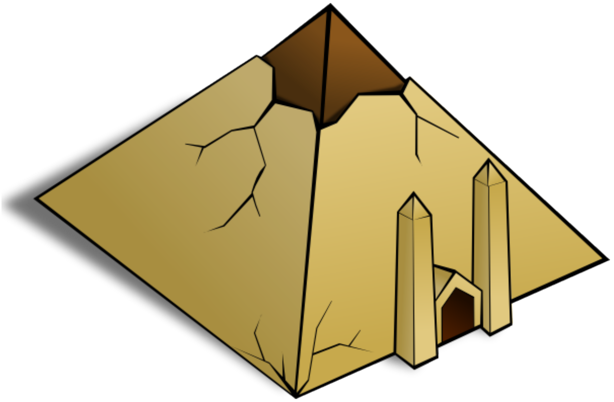

pig

choir

graduation

involuntary

The sun sets in the West.

The capable detective discovered why I’d been waiting.

Quick Aphasia Battery

Form 3

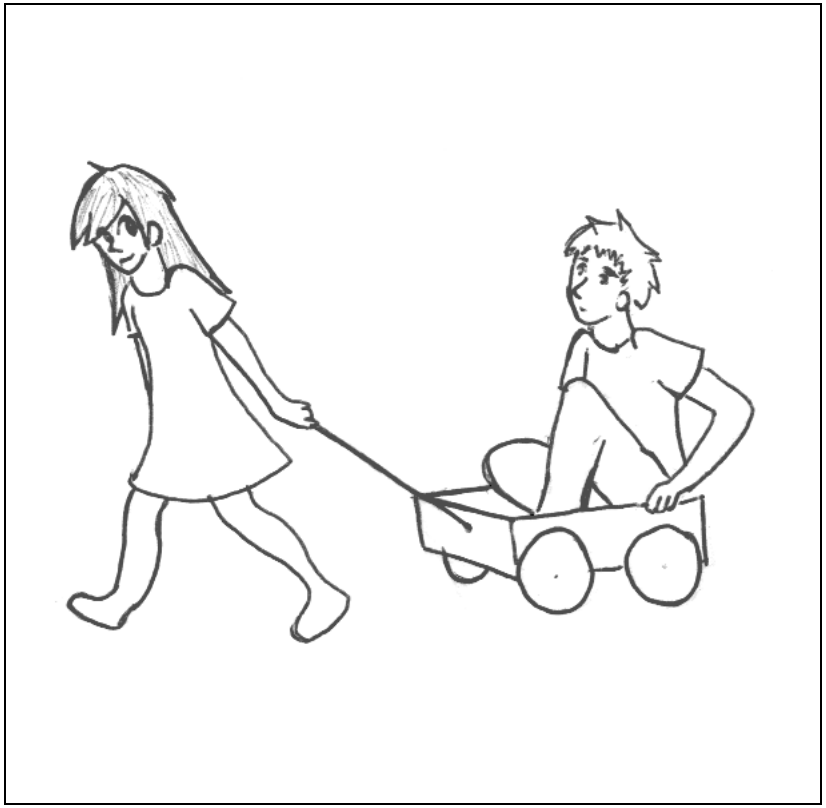

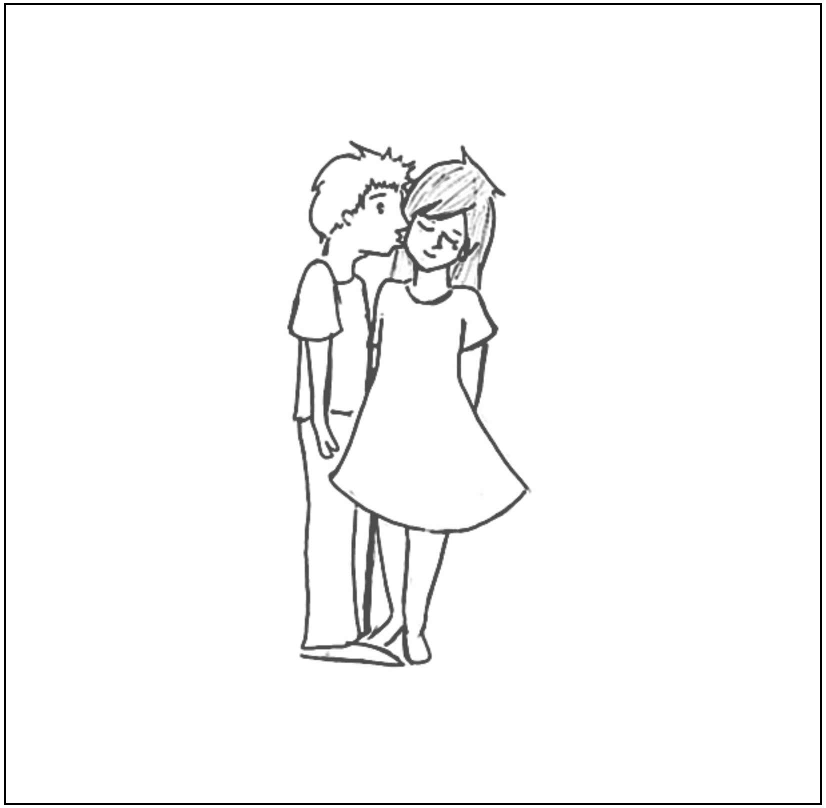

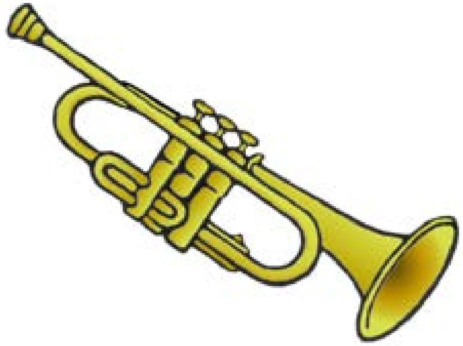
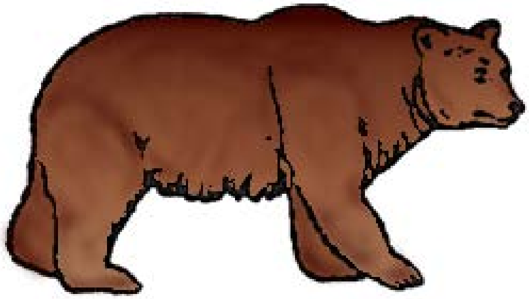
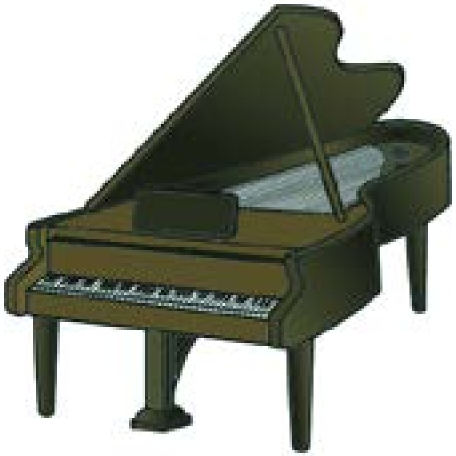
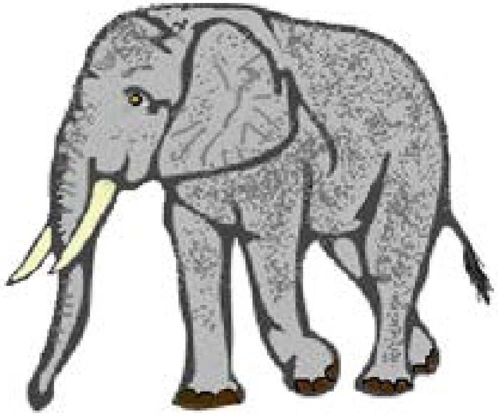
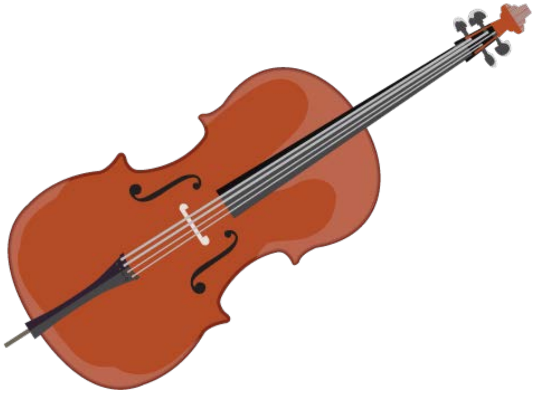
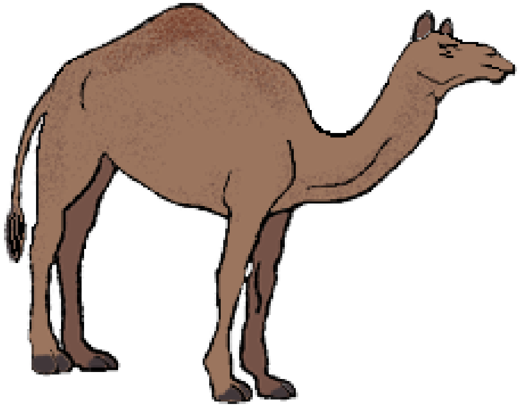

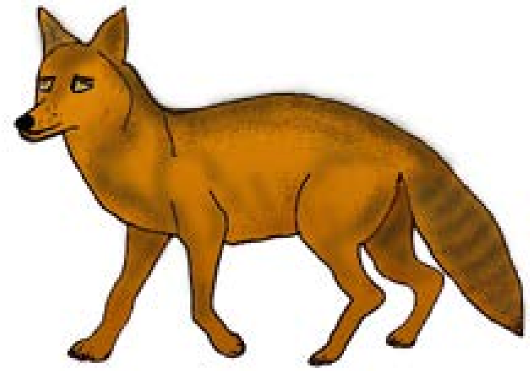
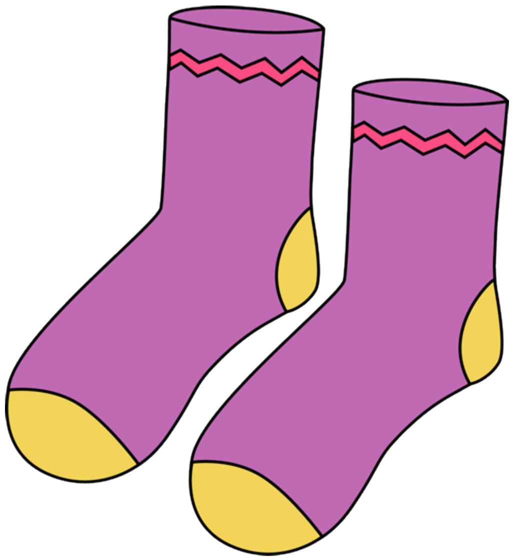
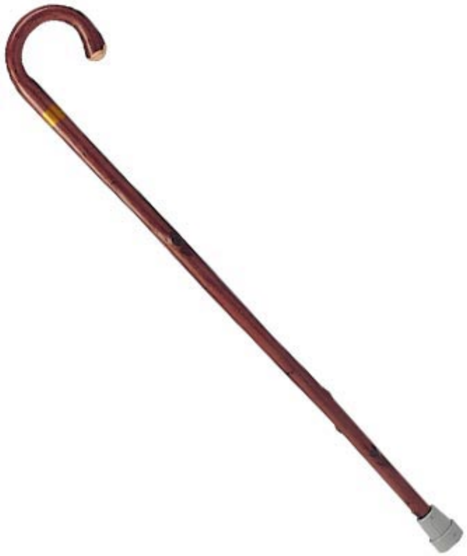
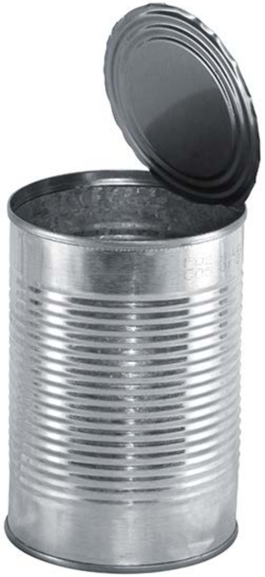
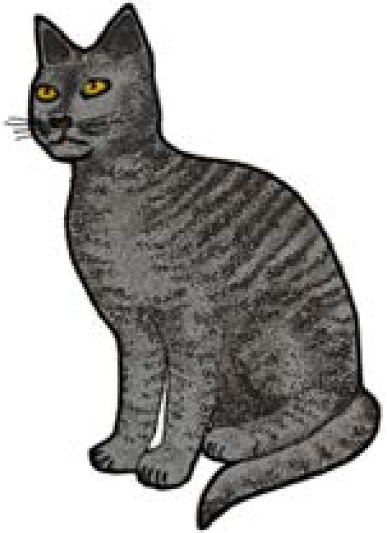
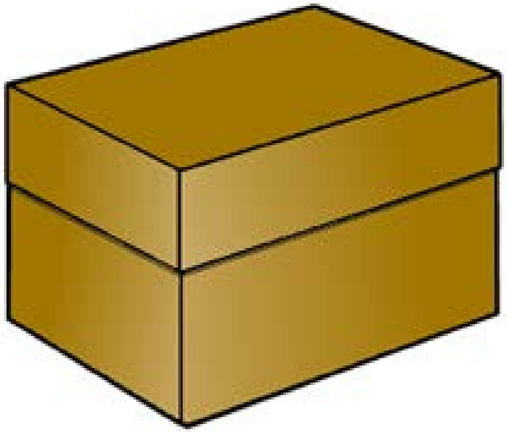

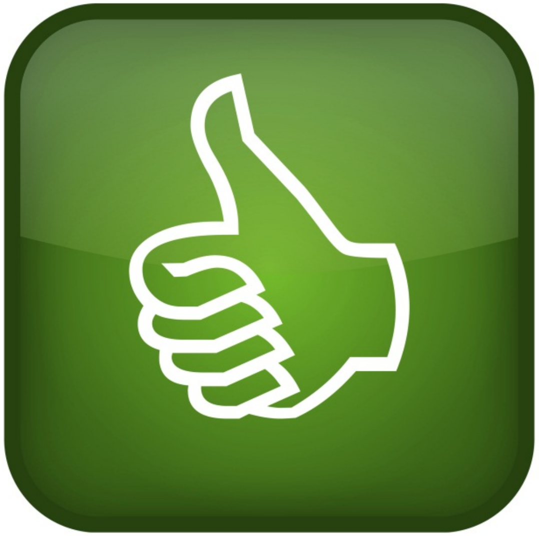

Yes

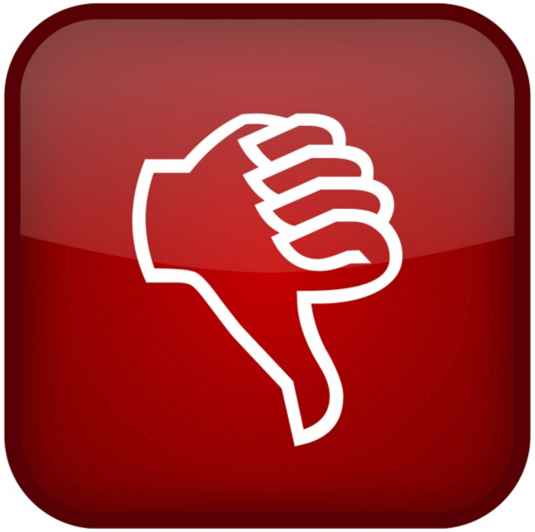

No

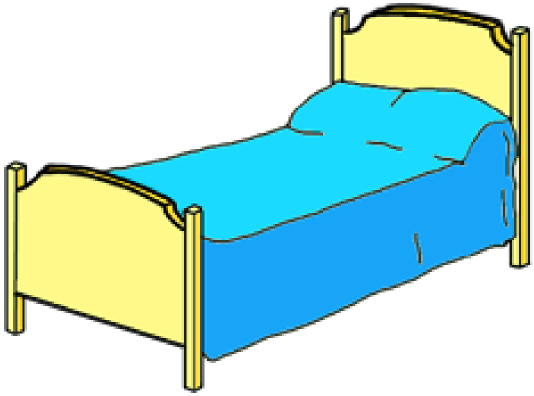

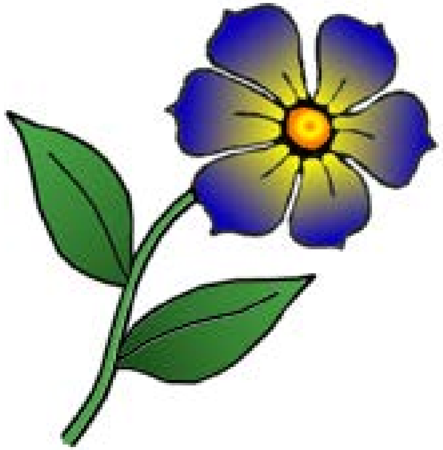

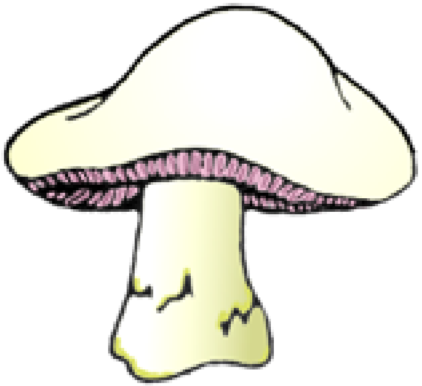

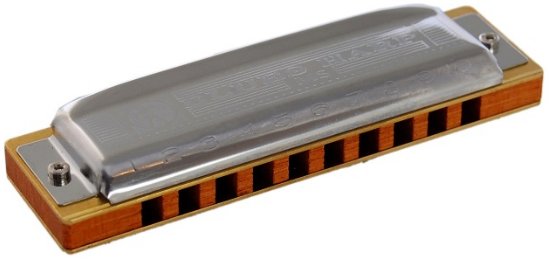

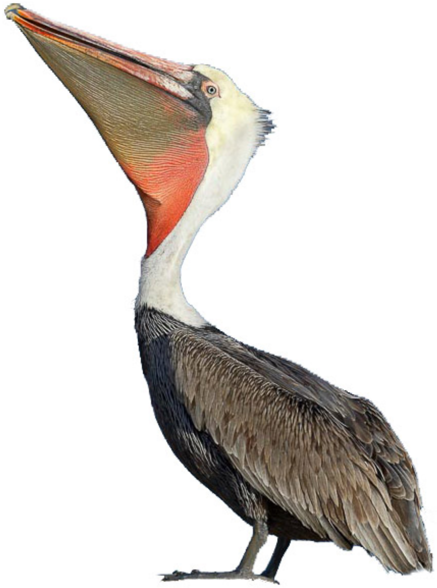

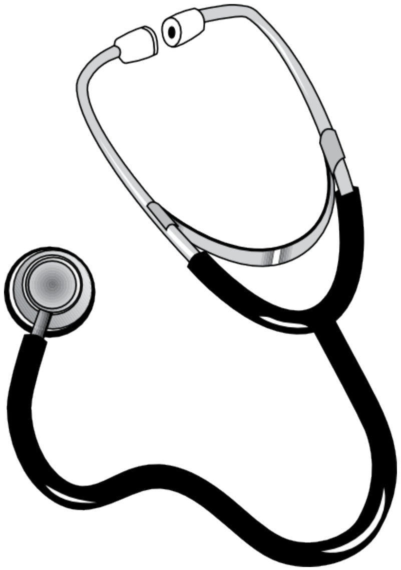

pot

cough

prohibition

unforgettable

The dog sleeps on the floor.

The ethical accountant understood why I’d been hiding.
